# The Salivary Healing Signature: From Disease Detection to Prediction and Monitoring of Periodontal and Peri-Implant Tissue Healing

**DOI:** 10.3390/diagnostics16203282

**Published:** 2026-10-09

**Authors:** Nada Tawfig Hashim, Bakri Gobara Gismalla, Muhammed Mustahsen Rahman, Rasha Babiker, Vivek Padmanabhan, Md Sofiqul Islam, Riham Mohammed, Mariam Elsheikh, Ayman Ahmed, Shadi El Bahra

**Affiliations:** 1 Department of Periodontics, RAK College of Dental Sciences, RAK Medical and Health Sciences University, Ras-AL Khaimah 12973, United Arab Emirates; 2 Department of Oral Rehabilitation, Faculty of Dentistry, University of Khartoum, Khartoum 11115, Sudan; 3 Department of Physiology, RAK College of Medical Sciences, RAK Medical and Health Sciences University, Ras Al Khaimah 11172, United Arab Emirates; 4 Department of Pediatric and Preventive Dentistry, RAK College of Dental Sciences, RAK Medical and Health Sciences University, Ras-Al Khaimah 12973, United Arab Emirates; 5 Department of Operative Dentistry, RAK College of Dental Sciences, RAK Medical and Health Sciences University, Ras-Al Khaimah 12973, United Arab Emirates; sofiqul.islam@rakmhsu.ac.ae; 6 Department of Oral Surgery, RAK College of Dental Sciences, RAK Medical and Health Sciences University, Ras-Al Khaimah 12973, United Arab Emirates; 7 Clinical Sciences Department, College of Dentistry, Ajman University, Ajman P.O. Box 346, United Arab Emiratess.elbahra@ajman.ac.ae (S.E.B.); 8 Department of Periodontology and Implantology, College of Dentistry, The National Ribat University, Khartoum 11115, Sudan; 9 Center for Medical and Bio-Allied Health Sciences Research, Ajman University, Ajman P.O. Box 346, United Arab Emirates

**Keywords:** salivary biomarkers, periodontitis, peri-implantitis, healing signature, treatment monitoring, point-of-care diagnostics, matrix metalloproteinases, inflammatory cytokines

## Abstract

Periodontal and peri-implant diseases cause progressive tissue destruction that may lead to tooth and implant loss. Conventional clinical and radiographic assessments document accumulated tissue damage but provide limited information on current biological activity or healing. This narrative review synthesizes the literature published between 2015 and 2025, identified through searches of PubMed/MEDLINE, Scopus, and Web of Science, on salivary biomarkers in periodontal and peri-implant diseases, with emphasis on diagnostic performance, changes after therapy, and clinical translation. Salivary inflammatory mediators and tissue- and bone-remodeling markers—including interleukin (IL)-1β, IL-6, IL-8, IL-17, tumor necrosis factor-α, matrix metalloproteinase (MMP)-8, MMP-9, calprotectin, and the RANKL/osteoprotegerin axis—are consistently associated with periodontal and peri-implant disease. Multimarker panels generally outperform single biomarkers, with some studies reporting areas under the curve above 0.90 for periodontitis detection; however, most evidence derives from cross-sectional diagnostic studies, and prospective prognostic data remain limited. We propose the “salivary healing signature” as a conceptual framework—not a validated test—in which serial multimarker measurements could complement clinical assessment for disease detection, risk stratification, and treatment monitoring. Standardized sampling and analytical protocols, control of confounders, defined thresholds, and prospective validation in diverse populations are required before clinical implementation.

## 1. Introduction

Periodontal diseases, encompassing gingivitis and periodontitis, affect a large proportion of adults worldwide—approximately half of adults in many epidemiological surveys, depending on the case definition used—and are a major cause of tooth loss in adults [1]. Peri-implant diseases—including peri-implant mucositis and peri-implantitis—have reported weighted mean prevalences of approximately 43% and 22%, respectively, although estimates vary widely with case definition, threatening the long-term success of dental implant therapy [2,3,4]. These inflammatory conditions share common pathophysiological mechanisms involving dysbiotic microbial communities, exaggerated host immune responses, and progressive destruction of supporting tissues. Beyond their local impact, periodontal and peri-implant diseases have been linked to systemic conditions including cardiovascular disease, diabetes mellitus, adverse pregnancy outcomes, and respiratory infections [5], underscoring their significance as major public health concerns.

Current diagnostic paradigms for periodontal and peri-implant diseases rely predominantly on clinical parameters such as probing depth, clinical attachment loss, bleeding on probing, and radiographic bone loss [6]. These measures remain the reference standard for diagnosis. Bleeding on probing, when probing is performed with appropriate force, provides information on current inflammation, and the 2017 classification of periodontal and peri-implant diseases incorporates stage, extent and distribution, and grade; grading uses direct or indirect evidence of past progression and risk factors such as smoking and diabetes to estimate the likely rate of future progression [7,8]. Nevertheless, attachment and bone loss largely document cumulative past tissue destruction, and the available clinical parameters have a limited ability to identify, at a single examination, which sites or patients are currently undergoing active breakdown or will respond poorly to therapy, or to describe the biological healing response after treatment [9,10]. Molecular biomarkers are therefore being investigated as adjuncts that may add information on current biological activity, rather than as replacements for clinical assessment [6,10].

The limitations of conventional diagnostics have stimulated research into molecular biomarkers that reflect disease activity and healing dynamics. Saliva has emerged as an attractive diagnostic biofluid for oral diseases, offering non-invasive collection, patient acceptability, and the presence of locally derived molecules from gingival crevicular fluid (GCF) that reflect periodontal tissue status [6,11,12]. Unlike serum biomarkers, which mainly reflect systemic inflammation, salivary biomarkers reflect the overall (whole-mouth) oral inflammatory burden; they do not provide site-specific information, which requires sampling of GCF or peri-implant crevicular fluid (PICF) [13,14]. Saliva is, however, readily accessible for repeated sampling during treatment monitoring [13,15].

In this review, the term “salivary healing signature” denotes a proposed conceptual framework rather than an existing or clinically validated diagnostic test. The concept moves beyond single biomarker detection toward multimarker profiling that attempts to capture the interplay of inflammation, tissue destruction, microbial dysbiosis, and regenerative processes [16,17]. Candidate components include inflammatory cytokines (IL-1β, IL-6, IL-8, IL-17, TNF-α), tissue degradation enzymes (MMP-8, MMP-9, neutrophil elastase), bone remodeling markers (RANKL, OPG, osteocalcin), antimicrobial peptides, microbial DNA signatures, and emerging omics-based biomarkers including microRNAs, metabolites, and extracellular vesicles [16,18,19,20]. To our knowledge, no signature with predefined biomarkers, weighting coefficients, decision thresholds, and independent external validation currently exists; the framework is offered to organize the current evidence and to define what such validation would require.

This narrative review synthesizes current evidence on salivary biomarkers for periodontal and peri-implant diseases. Numerous reviews have summarized salivary biomarkers for the detection of periodontitis; the present review differs in three respects. First, it focuses on the longitudinal dimension—how biomarker profiles change during and after therapy—and on the distinction between diagnostic, monitoring, prognostic, and predictive applications, which are often conflated. Second, it considers periodontal and peri-implant conditions in parallel, highlighting where the biomarker evidence is shared and where it diverges. Third, it explicitly considers the maturity of the evidence and the pre-analytical, biological, and methodological factors that currently limit clinical interpretation. We examine the mechanisms underlying biomarker release, evaluate the diagnostic accuracy of established and emerging biomarkers, describe temporal changes during healing, and assess the translational status of point-of-care technologies. Our aim is to provide a critical overview that may inform the design of future validation studies, rather than to propose biomarker-guided protocols for immediate clinical use.

## 2. Materials and Methods

### 2.1. Literature Search

This article was designed as a narrative (non-systematic) review. A structured literature search was conducted in PubMed/MEDLINE, Scopus, and Web of Science, supplemented by Google Scholar and by manual screening of the reference lists of relevant reviews for articles published between January 2015 and December 2025. Search terms combined disease-related terms (“periodontitis”, “periodontal disease”, “gingivitis”, “peri-implantitis”, “peri-implant mucositis”, “peri-implant disease”) with sample- and biomarker-related terms (“saliva”, “salivary”, “oral fluid”, “biomarker*”, “cytokine*”, “interleukin”, “matrix metalloproteinase”, “MMP-8”, “RANKL”, “osteoprotegerin”, “calprotectin”, “microbiome”, “proteomic*”, “metabolomic*”, “microRNA”, “extracellular vesicle*”) and with application-related terms (“diagnos*”, “accuracy”, “prognos*”, “predict*”, “monitoring”, “treatment outcome”, “healing”, “point-of-care”, “biosensor”, “machine learning”), using Boolean operators adapted to each database. The core PubMed search string was: (periodontitis OR “periodontal disease” OR peri-implantitis OR “peri-implant mucositis”) AND (saliva OR salivary OR “oral fluid”) AND (biomarker* OR cytokine* OR “matrix metalloproteinase*” OR microbiome OR proteom* OR metabolom* OR microRNA) AND (diagnos* OR prognos* OR predict* OR monitor* OR healing OR “point-of-care”).

### 2.2. Study Selection and Evidence Synthesis

English-language studies in humans were considered, including cross-sectional and case–control diagnostic studies, prospective and retrospective cohort studies, interventional studies reporting biomarker changes after periodontal or peri-implant therapy, technology-development studies of point-of-care devices, systematic reviews, meta-analyses, and consensus reports. Studies of GCF or PICF were included when they informed the interpretation of salivary findings. Case reports, conference abstracts, and studies without a periodontal or peri-implant outcome were excluded. Earlier seminal publications and consensus reports were additionally included where they provided essential context. Titles and abstracts were screened, and the full texts of potentially relevant articles were assessed by the authors; disagreements were resolved by discussion. Consistent with the narrative design, no formal risk-of-bias assessment or quantitative pooling was performed. Instead, findings were synthesized thematically, and the type of evidence underlying each statement—cross-sectional association, diagnostic accuracy, longitudinal monitoring, prognostic association, or prediction of response to a specific intervention—was distinguished according to the BEST (Biomarkers, EndpointS, and other Tools) biomarker categories [21].

### 2.3. Use of Generative Artificial Intelligence

During the preparation of this manuscript, the authors used Claude (Opus 5.5; Anthropic PBC, San Francisco, CA, USA) for language editing, restructuring of the text, and drafting of summary tables during the revision process. The authors reviewed and edited all output, verified the scientific content and references, and take full responsibility for the content of this publication.

## 3. Saliva as a Diagnostic Biofluid

Saliva represents a complex biological fluid containing over 3000 proteins, nucleic acids, lipids, and small molecules, many of which reflect the health status of oral and systemic tissues [15,22,23]. As a diagnostic specimen, saliva offers several advantages over traditional biofluids including blood and urine: collection is non-invasive, requiring only expectoration or passive drool; patients generally find salivary collection more acceptable than venipuncture; samples can be collected repeatedly for longitudinal monitoring; and collection can be performed outside clinical settings [11,22,23,24].

The diagnostic value of saliva for periodontal diseases stems from its unique composition, which reflects local oral inflammatory processes. Whole saliva is a mixture of secretions from the major salivary glands (parotid, submandibular, sublingual), minor salivary glands, and GCF—the serum transudate that flows through the gingival sulcus [14]. During periodontal inflammation, increased vascular permeability elevates GCF flow rates, delivering higher concentrations of locally produced inflammatory mediators, enzymes, and tissue breakdown products into the oral cavity [14]. Consequently, salivary concentrations of several host-derived biomarkers are higher in periodontitis than in periodontal health; however, the magnitude of the difference varies considerably among biomarkers, studies, populations, and assay platforms, and the distributions in patients and controls overlap substantially [25,26,27].

The source of salivary biomarkers is critical for interpreting their diagnostic significance. GCF-derived biomarkers most directly reflect periodontal tissue status and include inflammatory cytokines, proteolytic enzymes, bone remodeling markers, and bacterial products [14]. Salivary gland-derived components include mucins, immunoglobulins, and antimicrobial proteins that contribute to oral defense [13]. Distinguishing GCF-derived from gland-derived biomarkers is important for disease specificity; markers predominantly originating from GCF show stronger associations with clinical periodontal parameters [14].

Whole saliva versus crevicular fluid: Whole saliva is a pooled sample that integrates contributions from the salivary glands, the crevicular fluid of all teeth and implants, the oral mucosa, the tongue dorsum, and the oral microbiota. Periodontitis and peri-implantitis, in contrast, are site-specific diseases in which active breakdown may be confined to a small number of sites. A salivary biomarker concentration therefore reflects the whole-mouth inflammatory burden—broadly, a function of the number of affected sites, their inflammatory severity, and the volume of crevicular fluid they release, diluted by glandular secretion—rather than the activity of any individual site [13,14]. Several interpretive consequences follow: a few deep, actively progressing sites may be masked by an otherwise healthy dentition; generalized gingival inflammation may produce a signal similar to that of localized severe periodontitis; the number of teeth present and the salivary flow rate influence concentrations independently of disease activity; and, in partially dentate patients, salivary findings cannot attribute inflammation to implants rather than to natural teeth. GCF and PICF sampling provide site-level information but are technically demanding, time-consuming, and sensitive to sampling technique and blood contamination [14,28]. The two approaches are therefore complementary: saliva is better suited to patient-level screening, risk assessment, and longitudinal monitoring of the global inflammatory burden, whereas clinical examination and GCF/PICF analysis are required to localize disease. Throughout this review, statements about saliva refer to patient-level information unless otherwise specified.

Practical considerations for salivary collection profoundly affect biomarker measurements. Collection method (unstimulated whole saliva vs. stimulated saliva vs. GCF-specific collection) affects biomarker concentrations and composition [10,28]. Collection time should account for circadian variation, with morning collections after overnight fasting generally recommended [10]. Pre-collection restrictions including avoidance of food, drink, and oral hygiene procedures for 1–2 h minimize confounding variables [10]. Sample volume, processing time, centrifugation conditions, aliquoting, and storage temperature (−80 °C for long-term storage) must be standardized [10,28]. These analytical variables contribute substantially to inter-laboratory variability and have been a major barrier to clinical translation [28].

Biological and pre-analytical confounders: Beyond collection procedures, numerous patient-level factors influence salivary biomarker concentrations independently of periodontal or peri-implant status. Smoking is particularly important: it suppresses gingival bleeding and alters the levels of several inflammatory mediators so that biomarker thresholds derived from non-smokers may misclassify smokers [29,30]. Diabetes mellitus and the degree of glycemic control modify salivary inflammatory profiles and may reduce the discriminatory performance of biomarker panels developed in systemically healthy populations [31]. Age, sex and hormonal status (including pregnancy), obesity, and systemic inflammatory or autoimmune conditions may raise baseline inflammatory mediator levels, whereas medications—including non-steroidal anti-inflammatory drugs, corticosteroids, immunosuppressants, antibiotics, antiresorptive agents, and xerogenic drugs that reduce salivary flow—may suppress or concentrate specific analytes [10,20,28]. Other oral conditions (gingivitis, caries, endodontic or mucosal lesions, and removable prostheses), recent periodontal instrumentation or toothbrushing, blood contamination, and salivary flow rate further affect measured concentrations [10,28]. Because many studies do not report or adjust for these variables, published reference values and cut-offs should be interpreted cautiously. Future studies should standardize and report these factors, consider normalization strategies (e.g., biomarker output per minute or per total protein), and use stratified or multivariable analyses so that a change in a biomarker can be attributed to periodontal or peri-implant status rather than to confounding.

Beyond periodontal biomarkers, saliva contains indicators of systemic diseases including diabetes, cardiovascular disease, oral cancer, and infectious diseases, which has stimulated interest in its broader diagnostic use, although for most systemic conditions salivary tests remain investigational [15,22,23]. Advances in analytical technologies including multiplex immunoassays, mass spectrometry, next-generation sequencing, and biosensors have substantially expanded the repertoire of detectable salivary analytes and improved sensitivity to femtomolar concentrations [24,32]. The convergence of these technological advances with growing clinical evidence for salivary biomarker utility has increased interest in saliva as a diagnostic medium in dentistry and medicine [24].

## 4. The Salivary Healing Signature: Concept and Framework

The salivary healing signature is conceptualized as a dynamic, multi-dimensional molecular profile that captures the temporal evolution of inflammation, tissue destruction, microbial activity, and regenerative processes during periodontal and peri-implant disease and healing [16,17]. Unlike static biomarker measurements that provide a snapshot of disease status, the healing signature framework emphasizes longitudinal monitoring—tracking biomarker trajectories over time to distinguish normal healing responses from pathological processes [10,33].

Status of the concept: The salivary healing signature is a hypothesis-generating framework. It specifies which biological domains a clinically useful profile would probably need to capture and how these might be expected to change over time, but it is not a validated diagnostic or prognostic test. A validated signature would require (i) a predefined set of biomarkers measured with analytically validated assays; (ii) a prespecified algorithm with defined weighting coefficients; (iii) clinically meaningful thresholds or reference change values; (iv) demonstration of discrimination and calibration in independent external cohorts; and (v) evidence that its use improves clinical decisions or patient outcomes [28]. None of these steps has yet been completed for a periodontal or peri-implant healing signature, and the kinetic patterns described in this review should therefore be regarded as hypotheses to be tested prospectively.

**Figure 1:**
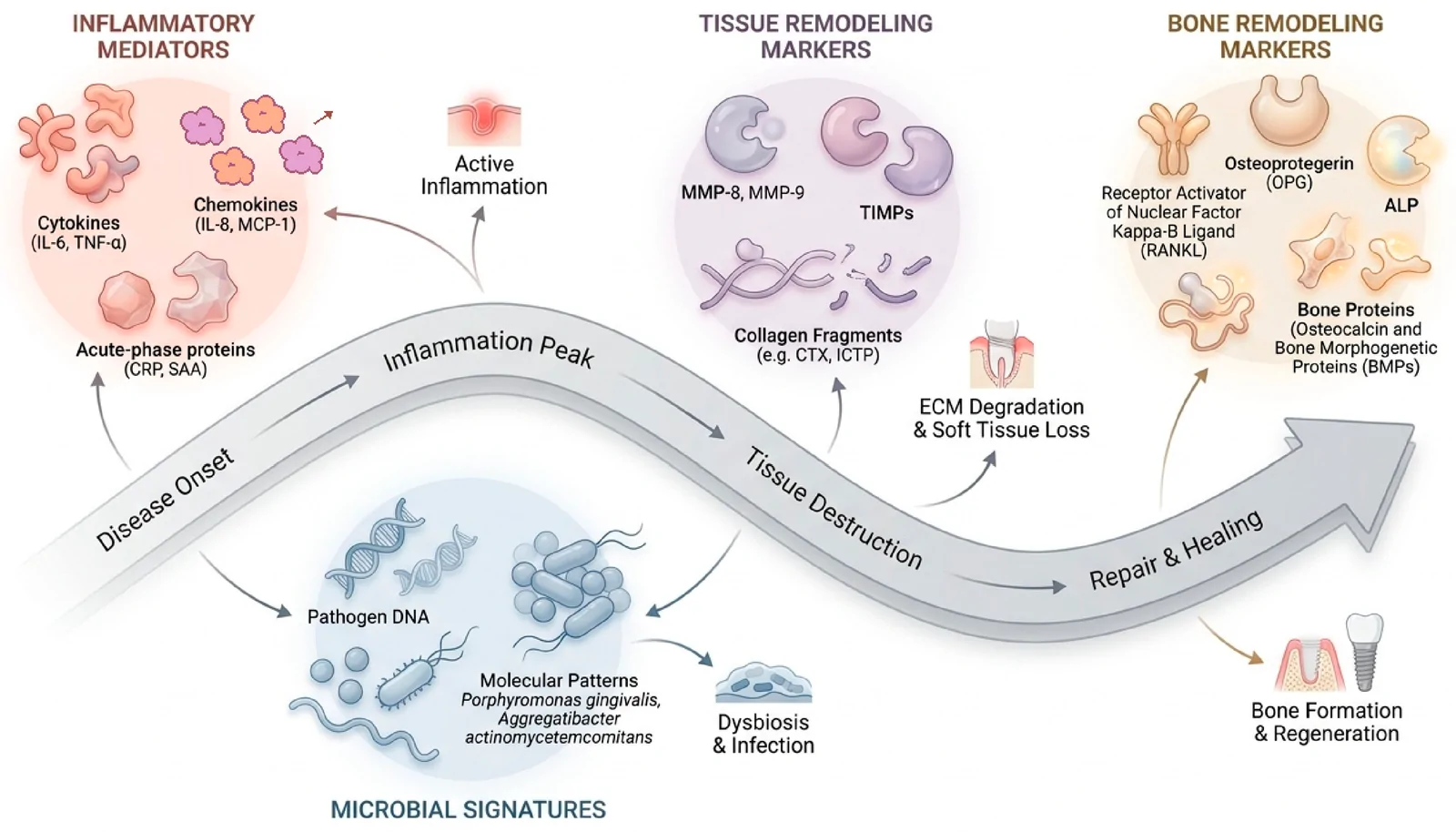
Biomarker dynamics during periodontal disease progression and healing. Microbial dysbiosis initiates an inflammatory response characterized by soluble mediators—cytokines, chemokines, and acute-phase proteins. Peak inflammation is followed by MMP/TIMP-mediated extracellular matrix and collagen degradation, reflecting soft-tissue destruction. Subsequent changes in the RANKL–RANK–OPG axis, ALP, osteocalcin, and BMPs reflect bone remodeling. Resolution of inflammation shifts the process toward tissue repair, bone formation, and regeneration The figure is a schematic, conceptual illustration; the sequence and relative timing of biomarker changes are idealized and are not drawn to a quantitative time scale. (Created with FigureLab v2.1).

The signature integrates four major biomarker categories. First, inflammatory mediators (cytokines, chemokines, acute-phase proteins) reflect the intensity and character of host immune responses [20,34]. Second, tissue remodeling markers (MMPs, their inhibitors, collagen degradation products) capture the balance between tissue destruction and repair [14,35]. Third, bone remodeling markers (RANKL, OPG, alkaline phosphatase, bone-specific proteins) reflect alveolar bone status and remodeling activity [36,37]. Fourth, microbial signatures (pathogen DNA, community composition, virulence factor activity) reflect the etiological driver of disease and its response to treatment [16,38] (Figure 1).

The diagnostic utility of the salivary healing signature rests on several key principles. Disease specificity may be enhanced by measuring multiple biomarkers reflecting different pathophysiological processes, reducing false positives from single-marker measurements [6,26,27]. Temporal resolution enables distinction between active disease, healing, and stable health states by tracking biomarker kinetics [33,39]. Patient-specific baselines account for inter-individual biological variation by comparing current levels to that patient’s own pre-treatment or healthy-phase values rather than population averages [10]. Risk stratification could use biomarker profiles to identify patients at elevated risk for progression or treatment failure, although this application has not yet been validated prospectively [10,40].

The conceptual framework builds upon decades of research identifying individual salivary biomarkers and now synthesizes these findings into an integrated diagnostic approach [6,10,40]. Multi-marker panels combining cytokines, MMPs, and bone remodeling markers generally outperform single biomarkers, with some studies reporting AUC values exceeding 0.90 for periodontitis detection compared to 0.70–0.85 for individual markers [25,26,27]; however, these estimates derive mostly from case–control designs comparing clearly defined disease and health groups, which tend to overestimate accuracy relative to the intended clinical population [26,27]. The ongoing incorporation of omics-based biomarkers (proteomics, metabolomics, microRNAs) and microbial signatures into comprehensive panels may further improve diagnostic accuracy and prognostic capability [41,42].

Implementation of the salivary healing signature requires analytical platforms capable of measuring multiple biomarkers simultaneously with sufficient sensitivity and specificity. Multiplex immunoassay platforms (e.g., Luminex^®^, electrochemiluminescence-based assays) enable quantification of 10–40 cytokines and growth factors in a single small sample [6]. Mass spectrometry-based proteomics provides comprehensive protein profiling [41]. PCR-based platforms enable simultaneous detection of multiple periodontal pathogens [38]. Point-of-care devices integrating biosensors with microfluidics are moving toward chairside multi-marker testing [43,44]. The optimal analytical platform for clinical implementation will depend on balancing cost, throughput, turnaround time, and analytical performance [43].

Biomarker categories according to intended use: A frequent source of overinterpretation in the salivary biomarker literature is the conflation of different types of claims. Following the BEST framework [21], we distinguish (i) diagnostic biomarkers, which detect or confirm the presence of disease (e.g., discriminating periodontitis from health); (ii) monitoring biomarkers, which are measured serially to assess disease status or response to therapy; (iii) prognostic biomarkers, which estimate the likelihood of a future clinical event—such as disease progression, recurrence, or tooth or implant loss—irrespective of treatment; (iv) predictive biomarkers, which identify individuals more likely to benefit from (or be harmed by) a specific intervention compared with an alternative, a claim that requires demonstration of a treatment-by-biomarker interaction, ideally in randomized comparisons; and (v) susceptibility/risk biomarkers, which indicate the potential for developing disease in currently healthy individuals. These categories require different study designs (Table 1). An association between a biomarker and disease severity in a cross-sectional study supports, at most, a diagnostic role; it does not establish that the same biomarker predicts treatment failure or implant loss. Most salivary evidence is diagnostic (cross-sectional or case–control), a smaller body addresses monitoring (pre- versus post-treatment changes), few studies are prospective prognostic studies, and we are not aware of any salivary biomarker validated as predictive in the strict sense. Accordingly, in this review, the terms “prognostic” and “predictive” are used in their strict sense, and statements that extend beyond the available study designs are identified as hypotheses.

## 5. Molecular Mechanisms of Biomarker Release

Understanding the cellular and molecular mechanisms underlying biomarker release into saliva is essential for interpreting biomarker data and developing targeted diagnostic strategies. Salivary biomarkers originate from multiple sources and enter saliva through distinct pathways, each reflecting different aspects of periodontal pathophysiology [14,15].

GCF-Derived Biomarkers: These represent the most direct reflection of periodontal tissue status. During inflammation, increased vascular permeability in the gingival connective tissue leads to enhanced GCF flow, carrying serum-derived proteins and locally produced inflammatory mediators into the gingival sulcus. The magnitude of GCF contribution to whole saliva depends on the extent and severity of periodontal inflammation: patients with generalized severe periodontitis have substantially higher GCF flow rates and consequently higher salivary biomarker concentrations than patients with localized mild disease [14].

Neutrophil Degranulation: A primary mechanism for release of tissue-destructive enzymes into periodontal tissues and saliva. Neutrophils are the predominant inflammatory cells in periodontal lesions, recruited in massive numbers in response to bacterial challenge [43,48]. Upon activation, neutrophils undergo degranulation, releasing preformed granule contents including MMP-8, MMP-9, elastase, lactoferrin, and calprotectin [14,43,48,49]. In chronic periodontal inflammation, excessive and prolonged neutrophil activation leads to collateral tissue damage [48].

Osteoclast Activity and Bone Remodeling: In periodontal disease, inflammatory cytokines (IL-1β, IL-6, IL-17, TNF-α) upregulate RANKL expression by osteoblasts and T cells, shifting the RANKL/OPG balance toward net bone resorption [20,36]. Activated osteoclasts secrete acid and proteases that dissolve bone mineral and degrade bone matrix proteins. Bone resorption products including collagen cross-links (ICTP, CTX) and bone matrix proteins (osteocalcin, osteopontin) are released into tissue fluids and can be detected in GCF and saliva [20,25].

Extracellular Vesicle Secretion: Extracellular vesicles (EVs), including exosomes and microvesicles, are membrane-bound nanoparticles secreted by virtually all cell types that carry proteins, lipids, and nucleic acids [16]. Salivary EVs have been shown to contain inflammatory mediators, microRNAs, and other biomarkers relevant to periodontal disease [16]. EVs may serve as stable carriers that protect biomarkers from degradation in the oral environment [16].

**Figure 2:**
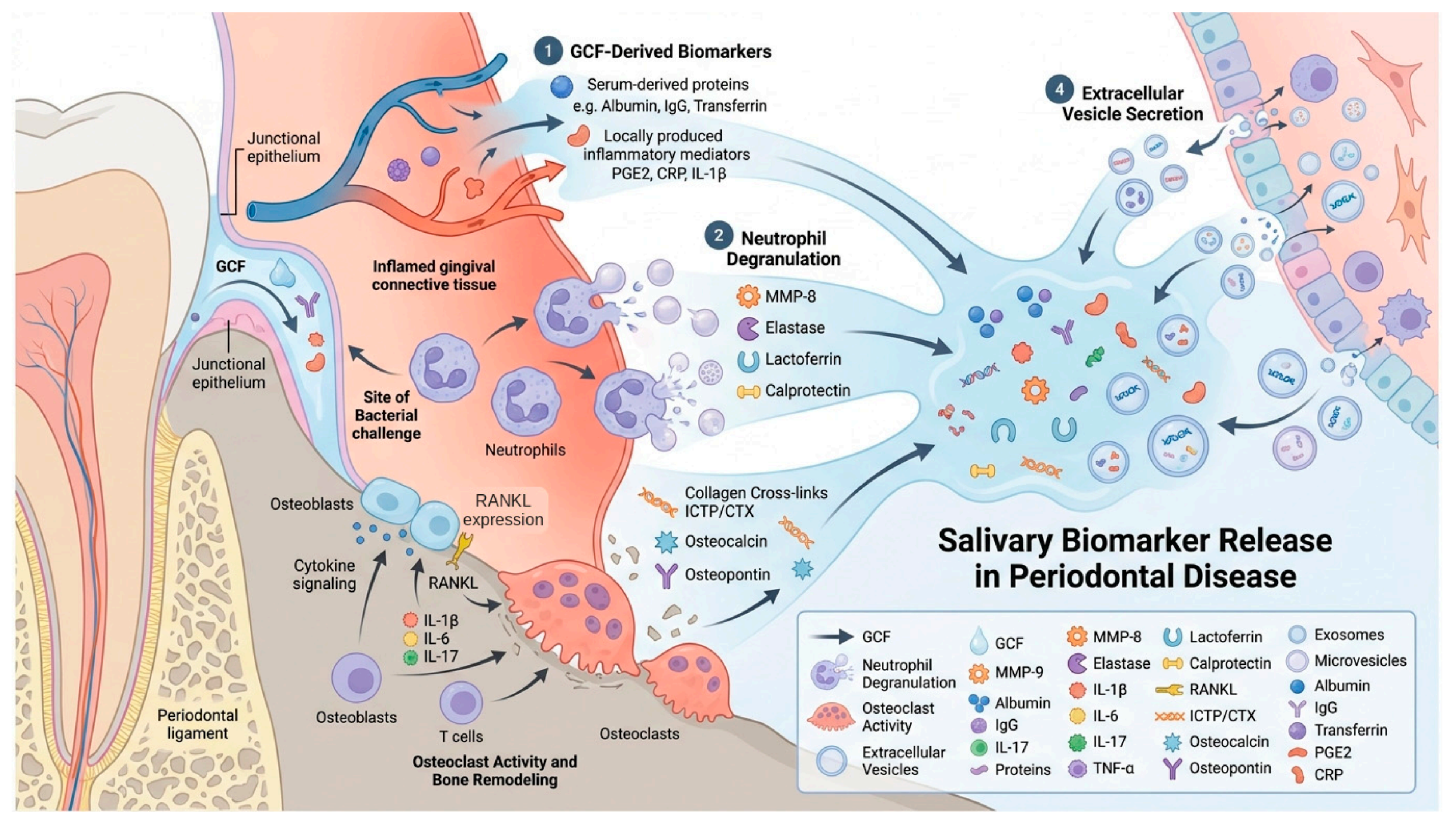
Salivary biomarker release in periodontal disease. Periodontal inflammation generates biomarkers from multiple local sources, including gingival crevicular fluid (GCF), inflammatory and immune cells, degradation of the periodontal connective tissue, and alveolar bone resorption. Cytokines and chemokines, matrix metalloproteinases (MMPs), neutrophil-derived enzymes, collagen-degradation products, and bone-turnover markers are released into the periodontal microenvironment and subsequently become detectable in whole saliva, principally through GCF and local tissue/cellular release. Extracellular vesicles may additionally transport biomolecular cargo derived from periodontal and inflammatory cells. Collectively, these salivary biomarkers provide measurable indicators of periodontal inflammation, connective-tissue destruction, and alveolar bone resorption. Schematic illustration; anatomical proportions are simplified. Created with FigureLab v2.1.

The combination of these release mechanisms creates a complex biomarker milieu in saliva that reflects the overall inflammatory burden and tissue status of periodontal and peri-implant tissues. Understanding the relative contributions of different sources and mechanisms enables more precise interpretation of salivary biomarker data and supports rational design of multi-marker diagnostic panels [6,14] (Figure 2).

## 6. Host-Derived Salivary Biomarkers

Inflammatory cytokines constitute the most extensively studied class of salivary biomarkers for periodontal and peri-implant diseases, reflecting the cellular immune responses that drive tissue destruction and modulate healing [20,34,38]. In line with the framework described in Section 4, host-derived biomarkers are presented below in three groups—inflammatory mediators (Section 6.1), tissue-remodeling markers (Section 6.2), and bone-remodeling markers (Section 6.3)—followed by microbial signatures (Section 7). Unless otherwise stated, the data in Section 6 and Section 7 refer to periodontitis; evidence for peri-implant diseases is presented separately in Section 8.

### 6.1. Inflammatory Mediators

**Figure 3:**
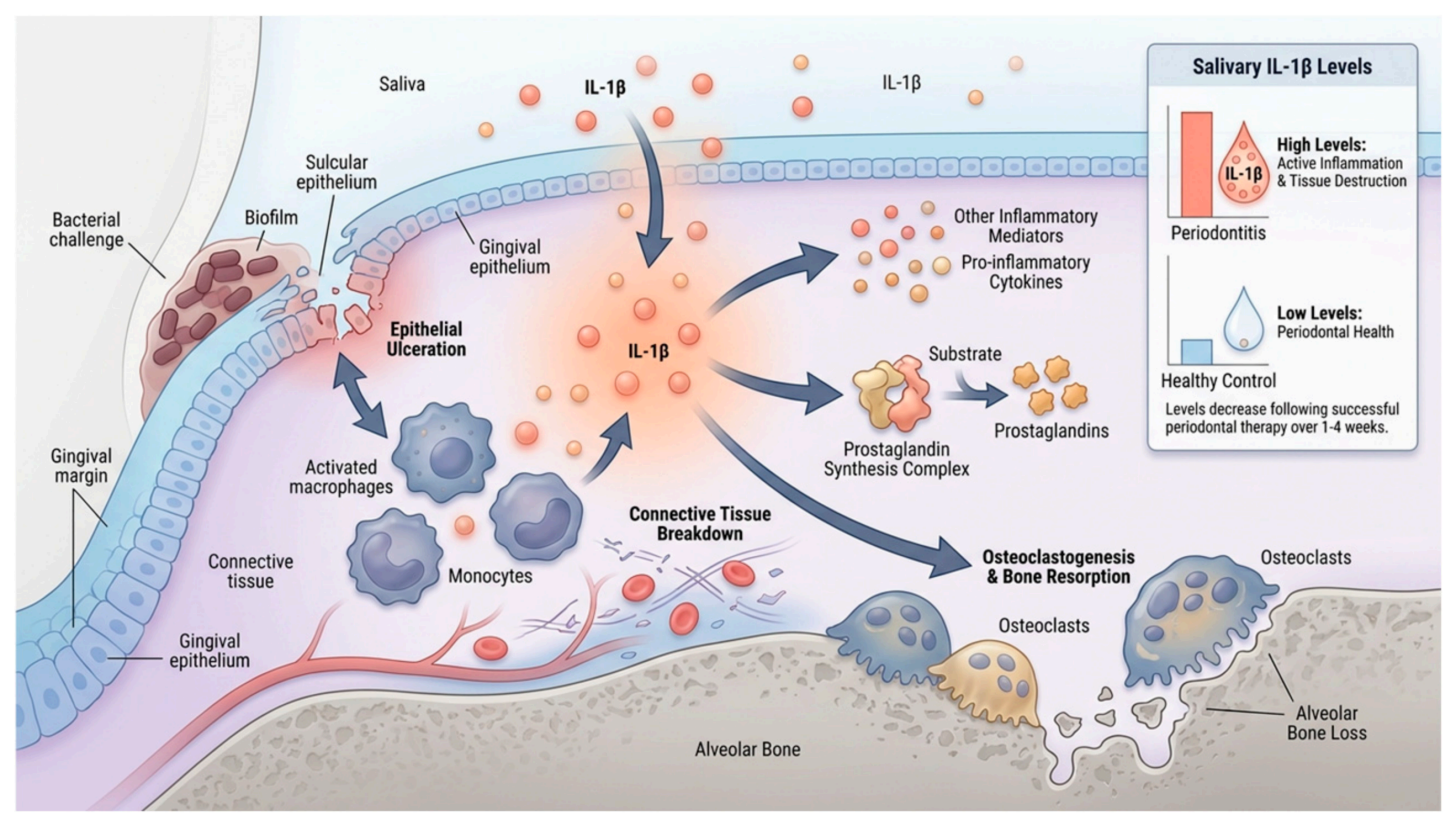
Role of salivary IL-1β in periodontal inflammation and bone destruction. Bacterial biofilm induces epithelial and immune-cell activation, promoting IL-1β release. Elevated IL-1β amplifies inflammatory mediator and prostaglandin production, contributing to connective-tissue breakdown, osteoclastogenesis, and alveolar bone resorption. Accordingly, increased salivary IL-1β may serve as a biomarker of active periodontal inflammation and tissue destruction (Created with FigureLab v2.1).

Interleukin-1β (IL-1β): This prototypical pro-inflammatory cytokine is produced predominantly by activated macrophages and monocytes in response to bacterial challenge. IL-1β is among the most consistently elevated and most extensively studied salivary biomarkers in periodontal disease; concentrations are several-fold higher in periodontitis than in periodontal health in most studies, although the reported magnitude varies widely with the study population, case definition, and assay [20,39,50,51]. IL-1β stimulates the production of other inflammatory mediators, promotes prostaglandin synthesis, activates osteoclastogenesis, and drives periodontal tissue destruction [39,51]. Diagnostic accuracy for distinguishing periodontitis from health is moderate to good, with AUC values typically in the range of 0.80–0.90 in systematic reviews [26,27], largely derived from case–control studies. IL-1β shows strong correlations with probing depth, clinical attachment loss, and bleeding on probing [50,51]. IL-1β levels have also been reported to decrease within weeks of successful non-surgical therapy, although this has been examined in relatively few longitudinal studies [39,50] (Figure 3).

Interleukin-6 (IL-6): A pleiotropic cytokine produced by macrophages, fibroblasts, and endothelial cells, IL-6 plays multiple roles in inflammatory responses including stimulation of acute-phase protein production, B-cell differentiation, and osteoclastogenesis [20,32,52]. Salivary IL-6 levels are elevated in periodontitis and correlate with disease severity [32,52]. IL-6 is frequently included in multi-marker panels for periodontal diagnosis and shows complementary diagnostic information to IL-1β [25,32]. In children with gingivitis, salivary IL-6 combined with IL-1β and CRP shows diagnostic utility [53].

Interleukin-8 (IL-8): Also known as CXCL8, IL-8 is a potent neutrophil chemoattractant produced by macrophages, epithelial cells, and fibroblasts [50,52]. IL-8 plays a central role in recruiting neutrophils to sites of infection, and its levels in GCF and saliva reflect the intensity of neutrophil infiltration in periodontal tissues [52]. Salivary IL-8 is elevated in periodontitis and shows strong correlations with clinical inflammation markers [50,52]. Studies have demonstrated that IL-8 levels decrease following periodontal therapy, paralleling clinical improvement [50].

Interleukin-17 (IL-17): A pro-inflammatory cytokine produced primarily by Th17 cells, IL-17 plays a critical role in host defense against extracellular bacteria [34,36,54]. IL-17 stimulates production of other inflammatory mediators, recruits neutrophils, and promotes osteoclastogenesis through upregulation of RANKL [34]. Its possible role in peri-implant disease is discussed in Section 8. A recent study found that salivary IL-17 combined with RANKL and OPG could differentiate periodontal health from disease [36]. IL-17 levels also show potential for monitoring treatment response [34,54].

Tumor Necrosis Factor-α (TNF-α): A master pro-inflammatory cytokine produced by activated macrophages, TNF-α plays central roles in inflammation, immune regulation, and bone resorption [20,50,52]. TNF-α stimulates production of other inflammatory mediators, upregulates adhesion molecules, induces apoptosis, and promotes osteoclast differentiation [55]. Salivary TNF-α levels are elevated in periodontitis patients and correlate with disease severity [20,52]. TNF-α has been included in multi-marker panels for periodontal diagnosis and shows promise for monitoring treatment outcomes [20,55].

### 6.2. Tissue-Remodeling Markers

Matrix Metalloproteinase-8 (MMP-8): Also known as collagenase-2 or neutrophil collagenase, MMP-8 is among the most extensively studied salivary biomarkers for periodontal disease [26,33,38,39,43]. MMP-8 is produced primarily by neutrophils and degrades type I collagen, the major structural protein of periodontal connective tissue and alveolar bone [14,35]. Elevated MMP-8 levels directly reflect active tissue destruction, supporting its use as a marker of collagenolytic activity [14,39]. The active form (aMMP-8) shows superior diagnostic accuracy compared to total MMP-8, specifically reflecting enzymatically active collagen degradation [56,57,58]. Salivary MMP-8 levels correlate strongly with clinical parameters including probing depth, attachment loss, and bone loss [14,25,39,50]. Point-of-care tests for aMMP-8 are commercially available and have been evaluated in clinical studies for chairside screening, although their accuracy varies with the population and case definition [56,57,58,59].

MMP-9, TIMPs, and other neutrophil-derived markers: MMP-9 (gelatinase B), released by neutrophils and macrophages, degrades denatured collagen and basement-membrane components and is frequently elevated in saliva in periodontitis together with MMP-8; the balance between MMPs and their tissue inhibitors, particularly TIMP-1, reflects net matrix turnover [35]. Calprotectin (S100A8/A9), an abundant neutrophil cytosolic protein, has been consistently identified in proteomic studies as elevated in periodontitis and correlates with disease severity [41,42,49]. Collagen degradation products released during matrix breakdown provide complementary information on tissue destruction (Section 6.3).

### 6.3. Bone-Remodeling Markers

RANKL/OPG Axis: The receptor activator of nuclear factor kappa-B ligand (RANKL) and its decoy receptor osteoprotegerin (OPG) are key regulators of osteoclast differentiation and bone resorption [20,36]. RANKL promotes osteoclastogenesis and bone resorption, while OPG inhibits these processes [20]. The RANKL/OPG ratio in saliva reflects the balance between bone resorption and formation, with elevated ratios indicating net bone loss [36,37]. Salivary RANKL and OPG levels have been investigated in both periodontal and peri-implant diseases, showing potential for assessing bone remodeling activity [13,36,37,60]; evidence that they predict implant outcomes remains limited (Section 8). Bone-turnover products, including the collagen cross-link telopeptides ICTP and CTX and bone matrix proteins such as osteocalcin and osteopontin, are released during osteoclastic resorption (Section 5) and have been measured in saliva; ICTP, for example, has been included with IL-1β and *P. gingivalis* in a multimarker panel [20,25]. Markers of bone formation, such as alkaline phosphatase, are discussed in relation to implant healing in Section 10.

The principal biomarkers, their sources, the predominant type of available evidence, and their main limitations are summarized in Table 2.

## 7. Microbial and Microbiome Signatures

While host-derived inflammatory biomarkers reflect the tissue-destructive consequences of periodontal disease, microbial biomarkers provide direct information about the etiological agents driving disease. Periodontal and peri-implant diseases are polymicrobial infections characterized by dysbiotic shifts in the oral microbiome [1,16,38].

Key Periodontal Pathogens: The “red complex” bacteria—*Porphyromonas gingivalis* (*P. gingivalis*), *Tannerella forsythia* (*T. forsythia*), and *Treponema denticola* (*T. denticola*)—are strongly associated with severe periodontitis and are frequently detected in saliva of periodontitis patients [25,38]. *P. gingivalis* is considered a keystone pathogen that disrupts host-microbe homeostasis through multiple virulence factors including gingipain proteases, fimbriae, and lipopolysaccharide [25,38]. Salivary detection of *P. gingivalis* DNA using PCR-based methods shows good diagnostic accuracy for periodontitis, with sensitivity and specificity exceeding 70% in most studies [25,38].

16S rRNA Gene Sequencing: This has substantially advanced the understanding of the oral microbiome by enabling culture-independent profiling of entire microbial communities [1,16]. Studies using 16S sequencing of subgingival plaque have generally shown that periodontitis is associated with a shift in community structure—enrichment of anaerobic Gram-negative and proteolytic taxa and depletion of health-associated commensals—often accompanied by higher species richness in subgingival samples [16,67]. Diversity findings in saliva are less consistent, with studies reporting higher, unchanged, or lower alpha-diversity in periodontitis; these discrepancies reflect differences in sampling site (saliva versus subgingival plaque), sequencing platform and targeted 16S region, bioinformatic pipeline and diversity metric, case definition and severity, and confounders such as smoking, diet, and antibiotic exposure [63]. Compositional shifts in specific taxa are therefore more informative than diversity indices alone. Salivary microbiome profiles can distinguish periodontitis patients from healthy controls with reasonable accuracy [16].

**Figure 4:**
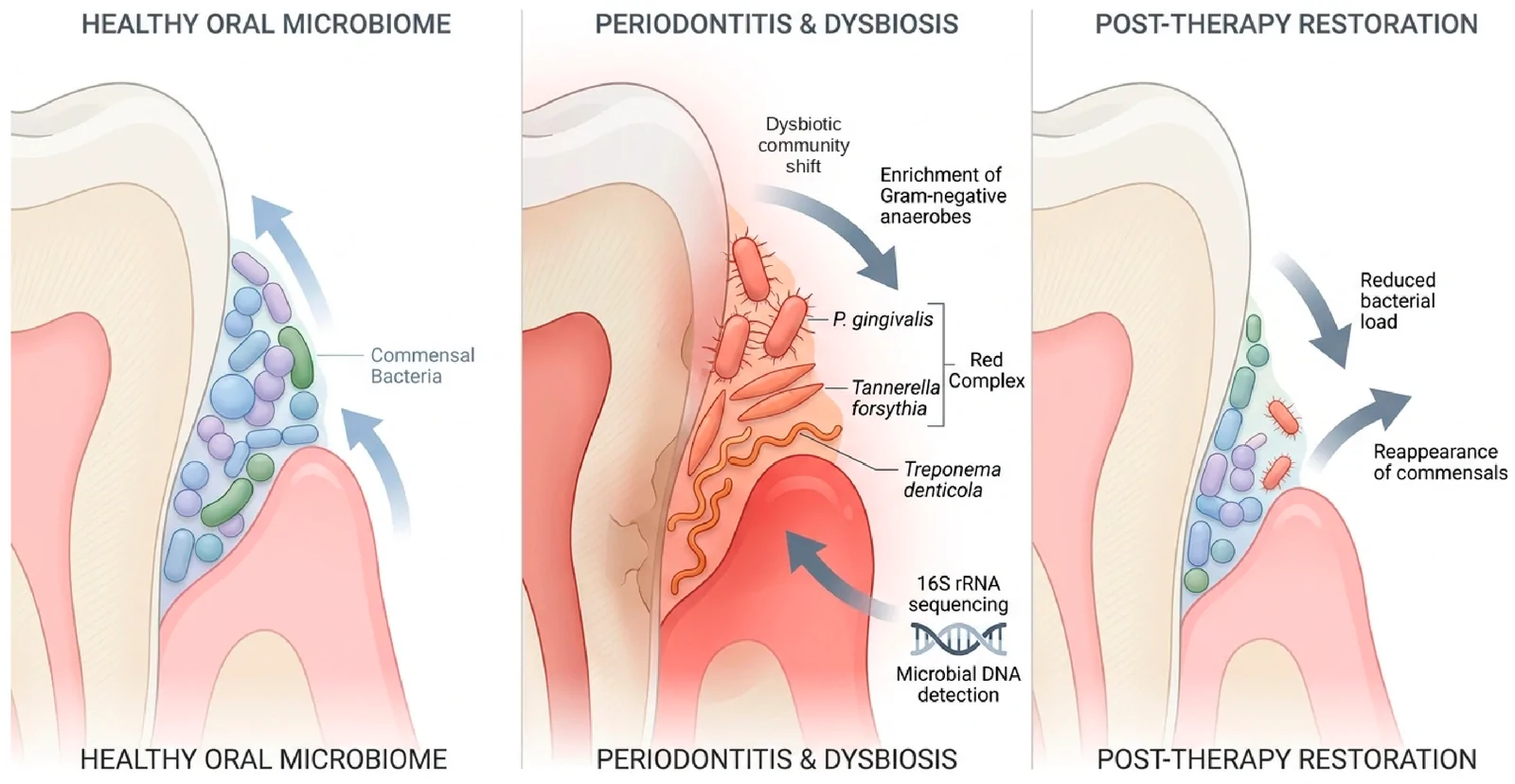
Microbial dysbiosis and restoration following periodontal therapy. Periodontal health is characterized by a balanced commensal microbiome, whereas periodontitis involves microbial dysbiosis, characterized by a shift in community structure with enrichment of Gram-negative anaerobic pathogens, including *P. gingivalis, T. forsythia,* and *T. denticola*. Periodontal therapy reduces the pathogenic bacterial burden and promotes re-establishment of a more health-associated microbial community Schematic illustration. (Created with FigureLab v2.1).

Salivary Microbiome Shifts with Treatment: Successful periodontal therapy produces characteristic microbiome changes: reduction in total bacterial load, decreased abundance of periodontal pathogens, and partial restoration of health-associated commensals [16]. Longitudinal monitoring of salivary microbiome composition during and after treatment may identify patients with inadequate microbial response who may require further clinical evaluation [16] (Figure 4).

Integration of microbial signatures with host-derived inflammatory biomarkers provides a more comprehensive picture of disease activity than either approach alone. Combined host-microbial panels incorporating pathogen detection, dysbiosis indices, and inflammatory markers have shown improved diagnostic accuracy compared with single-modality approaches in some studies [16,38].

## 8. Salivary Biomarkers in Peri-Implant Disease

Peri-implant diseases—peri-implant mucositis and peri-implantitis—represent inflammatory conditions affecting the soft and hard tissues surrounding dental implants, analogous to gingivitis and periodontitis around natural teeth [2,3]. While peri-implant diseases share pathophysiological features with periodontal diseases, including microbial dysbiosis and host inflammatory responses, important differences exist in tissue architecture, immune responses, and healing capacity that may be reflected in distinct biomarker signatures [2,3,13].

Peri-implant mucositis: According to the 2017 World Workshop, peri-implant mucositis is characterized by inflammation of the peri-implant mucosa—bleeding and/or suppuration on gentle probing—without bone loss beyond initial bone remodeling, and it is considered reversible [68].

Peri-implantitis: Peri-implantitis is a plaque-associated pathological condition occurring in the tissues around dental implants, characterized by inflammation of the peri-implant mucosa and subsequent progressive loss of supporting bone [68]. Reported weighted mean prevalence estimates are approximately 43% for peri-implant mucositis and 22% for peri-implantitis, although estimates vary widely with the case definition applied [3,4]. Early detection of peri-implant disease is clinically important, as advanced peri-implantitis is difficult to treat and often results in implant failure [3,69].

Similarities with periodontitis biomarkers: Many inflammatory biomarkers elevated in periodontitis are also elevated in peri-implant diseases. Salivary IL-1β, IL-6, TNF-α, and MMP-8 levels are consistently elevated in peri-implantitis compared to healthy implants [3,13,69,70]. A systematic review identified 49 different biomarkers investigated in peri-implant diseases, with IL-1β, RANKL, IL-6, TNF-α, and MMP-8 showing significantly higher levels in peri-implantitis compared to healthy implants [3]. These shared biomarkers reflect common inflammatory and tissue-destructive mechanisms in both conditions [3,13].

**Figure 5:**
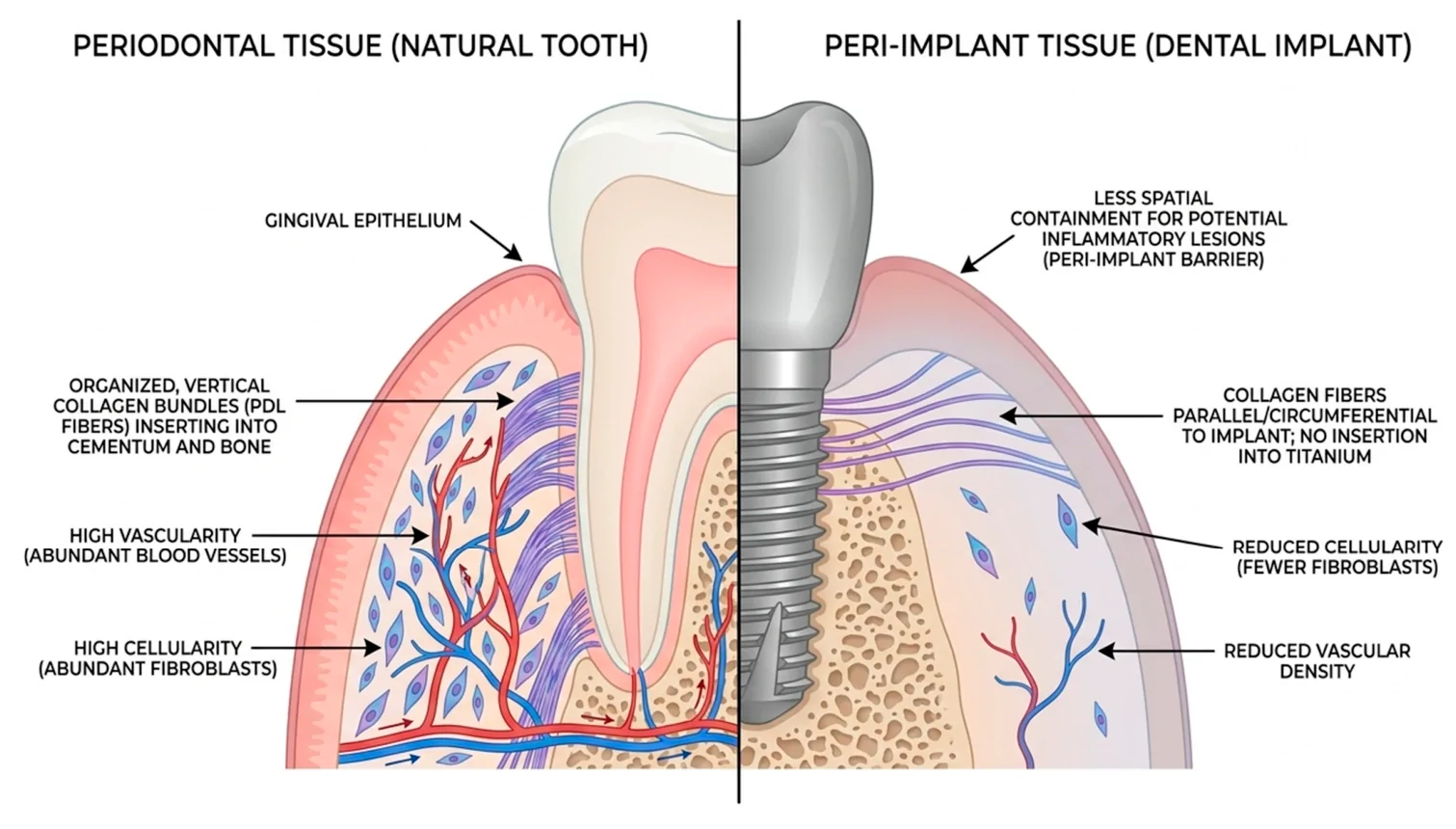
Structural differences between periodontal and peri-implant soft tissues. Periodontal tissues exhibit organized collagen fiber bundles inserting into cementum and alveolar bone, together with greater fibroblast cellularity and vascularity supported by the periodontal ligament. In contrast, peri-implant connective tissue contains predominantly parallel/circumferential collagen fibers without insertion into the implant surface, fewer fibroblasts, and reduced vascular density. These structural differences contribute to a comparatively less vascular and less spatially contained peri-implant soft-tissue barrier, potentially facilitating apical extension of inflammation during peri-implant disease Schematic illustration (Created with FigureLab v2.1).

Differences from periodontitis: Despite similarities, important differences exist between periodontal and peri-implant biomarker profiles. Peri-implant tissues lack the periodontal ligament and cementum present around natural teeth, resulting in different tissue architecture and potentially altered inflammatory responses [2,13]. The peri-implant connective-tissue interface differs fundamentally from the periodontal attachment apparatus. Around natural teeth, collagen fiber bundles insert into cementum and alveolar bone through the periodontal ligament (PDL), creating a highly organized, vascularized and cellular attachment system. In contrast, peri-implant collagen fibers are predominantly oriented parallel or circumferentially to the implant surface and do not insert into titanium. The peri-implant supracrestal connective tissue also has relatively fewer fibroblasts and vascular structures than periodontal tissues, partly because an implant lacks the vascular contribution normally provided by the PDL. Consequently, the peri-implant soft-tissue barrier has a different structural and reparative environment and may be less capable of spatially containing an advancing inflammatory lesion (Figure 5).

**Figure 6:**
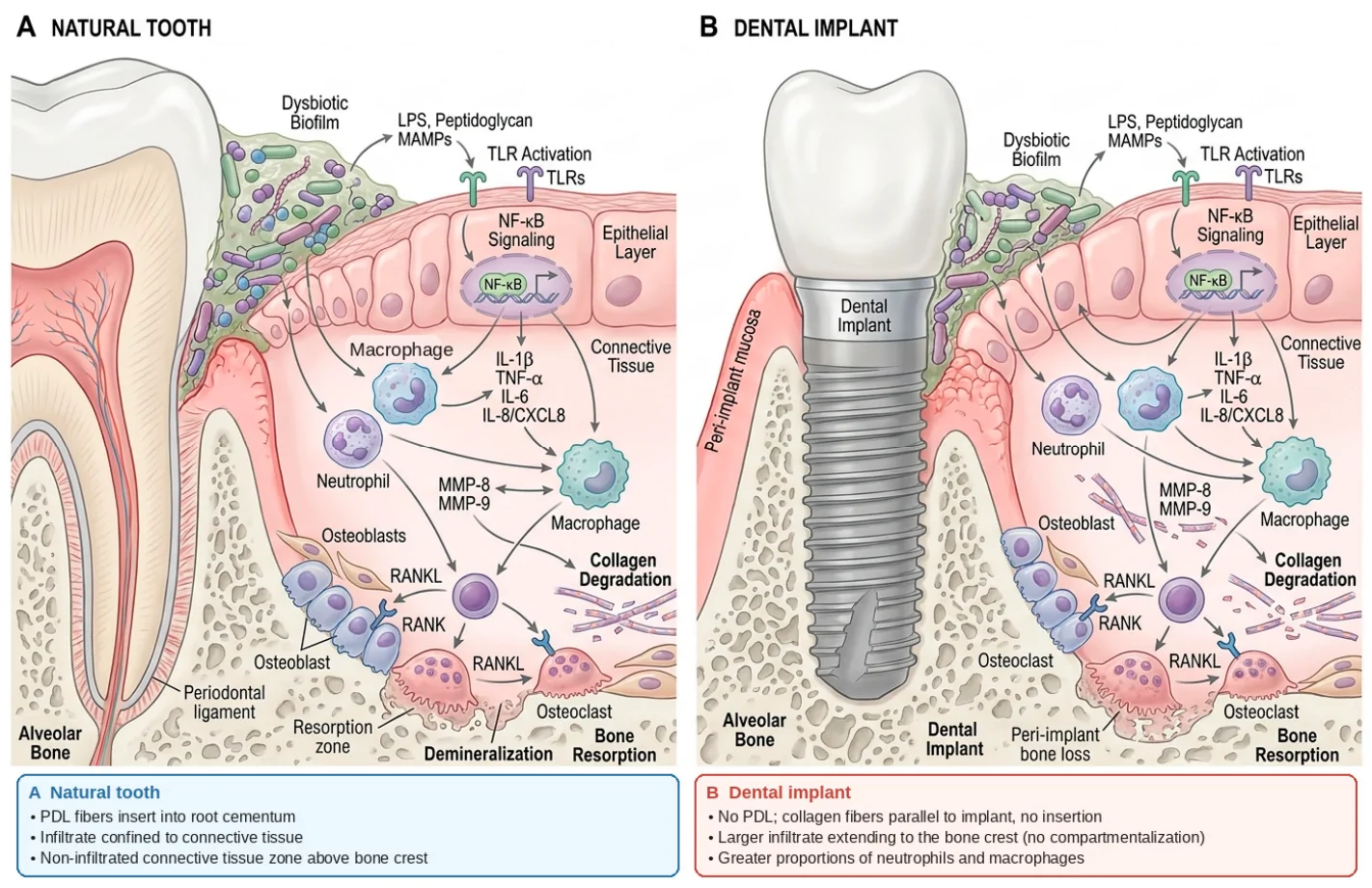
Comparative molecular pathways of tissue destruction in periodontitis (**A**) and peri-implantitis (**B**). Dysbiotic biofilms activate epithelial pattern-recognition receptors, including TLRs, through microbial products such as LPS and peptidoglycan, triggering NF-κB signaling and release of IL-1β, TNF-α, IL-6, and chemokines. Recruitment and activation of neutrophils and macrophages promote MMP-8/MMP-9-mediated collagen degradation, while increased RANKL–RANK signaling drives osteoclastogenesis and bone resorption. These pathways are largely shared; the principal differences are indicated in the boxes beneath each panel. Around the natural tooth (**A**), periodontal ligament fibers insert into root cementum and the inflammatory infiltrate is confined to the connective tissue, separated from the alveolar bone crest by a zone of non-infiltrated connective tissue. Around the implant (**B**), there is no periodontal ligament and no fiber insertion into the implant surface, and the infiltrate is larger, extends to the bone crest without connective-tissue compartmentalization, and contains greater proportions of neutrophils and macrophages [71] (Created with FigureLab v2.1).

At the molecular level, microbial products associated with dysbiotic biofilms activate pattern-recognition receptors, particularly Toll-like receptor pathways, on epithelial cells, neutrophils, macrophages and other resident cells. Downstream signaling converges on transcriptional pathways such as NF-κB, promoting production of IL-1β, TNF-α, IL-6, IL-8/CXCL8 and other chemokines. These mediators recruit and activate additional neutrophils and macrophages and stimulate production and activation of matrix metalloproteinases, particularly MMP-8 and MMP-9, contributing to collagen and extracellular-matrix degradation. At the bone interface, inflammatory cytokines favor increased RANKL-mediated RANK signaling, promoting osteoclast differentiation and activation and thereby linking soft-tissue inflammation to peri-implant bone resorption. These pathways are not unique to peri-implantitis—they are also central to periodontitis—but their magnitude and spatial organization may differ [13,71] (Figure 6).

**Figure 7:**
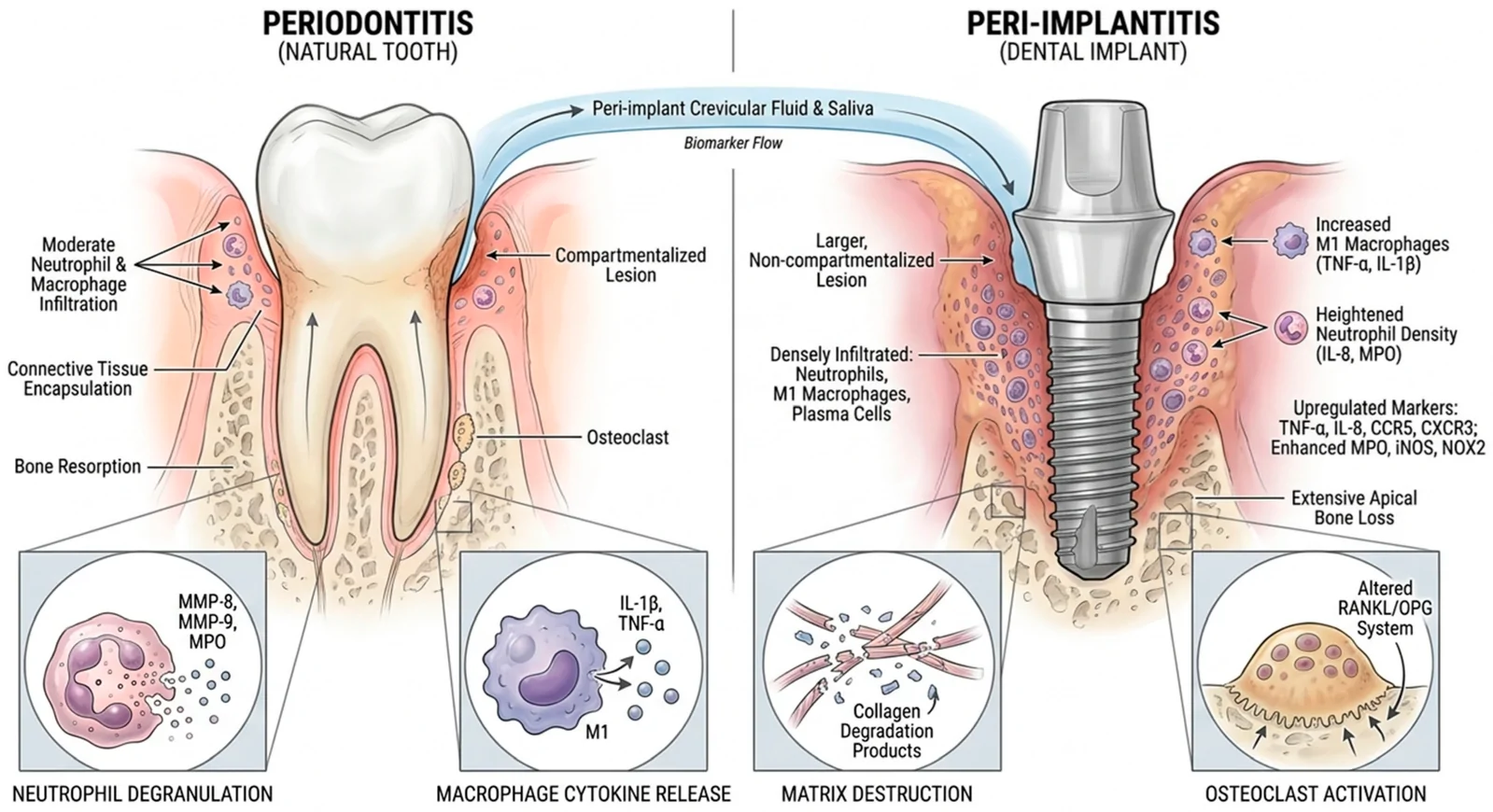
Comparative inflammatory and molecular characteristics of periodontitis and peri-implantitis. Both conditions share neutrophil/macrophage activation, pro-inflammatory cytokine release, MMP-mediated matrix degradation, and RANKL-driven osteoclastogenesis. However, peri-implantitis is characterized by a larger, less compartmentalized inflammatory lesion with greater neutrophil and macrophage infiltration and enhanced inflammatory and oxidative activity. These changes may increase MMP-8/MMP-9, MPO, IL-1β, TNF-α and related mediators, promote collagen degradation and RANKL/OPG-associated osteoclast activation, and contribute to more extensive apical bone loss. Released inflammatory and tissue-breakdown products can enter peri-implant crevicular fluid and saliva, providing potential biomarkers of disease activity (Created with FigureLab v2.1).

Importantly, histopathological evidence indicates that peri-implantitis should not simply be described as “periodontitis around an implant.” Peri-implantitis lesions are generally larger and extend further apically, often approaching the bone crest without the connective-tissue compartmentalization observed around teeth [71]. They also contain greater densities of neutrophils and macrophages, together with abundant plasma cells. Increased M1 macrophage polarization and higher expression of selected inflammatory transcripts, including TNF-α, IL-8, CCR5 and CXCR3, have also been reported in comparative studies. Enhanced MPO, iNOS and NOX2-associated oxidative/nitrosative activity further supports an amplified innate inflammatory environment in peri-implantitis. This difference has an important consequence for biomarker interpretation. Increased neutrophil activity may enhance the release of MMP-8, MMP-9, MPO and other granule-derived molecules; macrophage activation contributes cytokines such as IL-1β and TNF-α; extracellular-matrix destruction releases collagen-derived degradation products; and osteoclastogenesis alters bone-turnover mediators such as the RANKL/OPG system. These molecules can enter peri-implant crevicular fluid and subsequently saliva. Thus, elevated salivary or crevicular biomarkers in peri-implantitis may represent the combined molecular output of microbial challenge → innate immune amplification → matrix degradation → osteoclast activation, rather than simply reflecting the presence of inflammation (Figure 7).

A key scientific qualification is needed, however: no cytokine or microbial signature has yet been established as uniquely specific for peri-implantitis. The 2017 World Workshop concluded that no unique pro-inflammatory cytokine profile has been identified, and more recent evidence supports substantial biological overlap between periodontitis and peri-implantitis. The most defensible interpretation is therefore that peri-implantitis represents a quantitatively amplified and anatomically less-contained expression of largely shared inflammatory pathways, rather than a completely distinct molecular disease [13,68,72].

Peri-Implant Microbiome: Peri-implantitis is associated with enrichment of periodontal pathogens including *P. gingivalis*, *T. forsythia*, and *T. denticola*, but also shows unique features including higher abundance of Staphylococcus species and other facultative anaerobes [61,62]. Salivary microbiome profiling can distinguish peri-implantitis from healthy implants, with specific taxa showing strong associations with peri-implant disease [61,62].

IL-17 in peri-implant disease has been proposed as a mediator of particular interest because of its role in RANKL upregulation and osteoclast activation [34,36]. However, salivary IL-17 data in peri-implant disease remain limited, and studies combining IL-17 with RANKL and OPG have so far been conducted mainly in periodontitis [36,37]; whether this combination can distinguish healthy implants from peri-implantitis or predict implant outcomes requires investigation.

Galectin-3, a β-galactoside-binding lectin involved in inflammation and immune regulation, has been investigated as a potential biomarker for peri-implant disease, though evidence remains limited [3]. Galectin-3 may play roles in macrophage activation and fibrosis in peri-implant tissues [3].

Titanium ions and particles released from implant surfaces during mechanical loading or corrosion may trigger inflammatory responses and contribute to peri-implantitis pathogenesis [2]. Detection of titanium in peri-implant tissues and fluids has been reported, though the utility of titanium measurement as a salivary biomarker remains unclear [2]. Titanium particles may activate macrophages and promote inflammatory cytokine production, potentially contributing to the biomarker signature of peri-implantitis [2].

**Figure 8:**
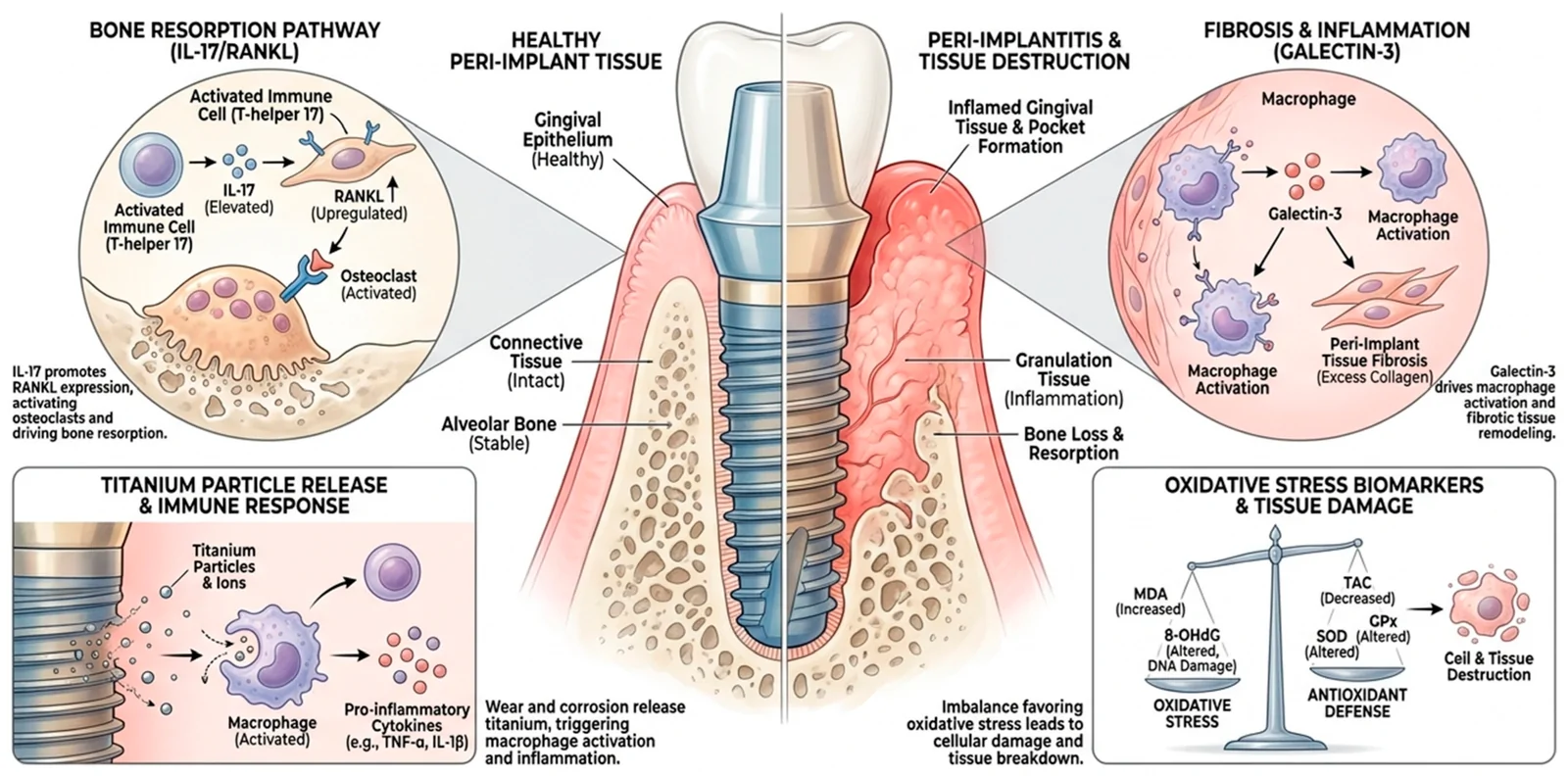
Molecular mechanisms of peri-implant tissue destruction. Peri-implantitis involves interconnected pathways of inflammation, oxidative stress, extracellular-matrix remodeling, and osteoclast-mediated bone resorption. Pro-inflammatory cytokines promote RANKL–RANK signaling and osteoclastogenesis, while macrophage activation and MMP activity contribute to collagen and matrix degradation. Titanium particle release may further stimulate innate immune responses. Increased oxidative stress, together with impaired antioxidant defenses, can amplify cellular and tissue injury, whereas persistent inflammation may promote fibrotic remodeling through mediators such as galectin-3. Collectively, these pathways contribute to progressive peri-implant soft-tissue destruction and marginal bone loss (Created with FigureLab v2.1).

Oxidative stress biomarkers have also been investigated in peri-implant disease. A systematic review and meta-analysis found that salivary malondialdehyde (MDA), a marker of lipid peroxidation, is significantly elevated in peri-implant disease, while total antioxidant capacity (TAC) is reduced [64,65]. Other oxidative stress markers including 8-hydroxydeoxyguanosine (8-OHdG), superoxide dismutase (SOD), and glutathione peroxidase (GPx) show altered levels in peri-implantitis [64,65]. These findings suggest that oxidative stress may play a role in peri-implant tissue destruction and that oxidative biomarkers may complement traditional inflammatory markers for peri-implant disease diagnosis [64,65] (Figure 8).

Novel biomarkers for peri-implantitis: Recent research has identified several novel salivary biomarkers with potential specificity for peri-implant disease. CXCL14 (a chemokine) and miR-4484 (a microRNA) together distinguished peri-implantitis from healthy implants with 100% accuracy in a single small study; such estimates are likely to be optimistic and require independent replication [66]. Pentraxin-3, an acute-phase protein, showed the highest diagnostic potential (AUC = 0.74) among inflammatory mediators for distinguishing peri-implantitis from health [72]. Procalcitonin, typically associated with bacterial infections, has been detected at elevated levels in saliva of peri-implantitis patients [73,74]. These emerging biomarkers require validation in larger studies but may offer improved specificity for peri-implant disease compared to traditional inflammatory markers.

Treatment monitoring in peri-implant disease: Salivary biomarkers may be useful for monitoring response to peri-implant therapy. Following surgical treatment of peri-implantitis, salivary levels of sTREM-1 and MMP-8 decrease significantly, paralleling clinical improvement [45]. MMP-8 has been evaluated as an adjunctive biomarker for monitoring peri-implant disease treatment, with point-of-care tests available for chairside use [45,57,58]. The time course of these changes is discussed in Section 10.

## 9. Prognostic and Predictive Biomarkers for Treatment Outcomes

Identifying, before or early after treatment, which patients are likely to respond favorably and which are at risk of non-response or disease progression would be among the most clinically valuable applications of salivary biomarkers [6,20,32]. However, as outlined in Section 4, most available studies report associations between baseline or post-treatment biomarker levels and clinical outcomes (prognostic or monitoring information), and none has demonstrated that a salivary biomarker identifies differential benefit from a specific intervention. The applications described below should therefore be regarded as potential uses that require prospective validation [10,40].

Pre-treatment baseline factors: Higher baseline IL-1β, MMP-8, or other inflammatory cytokine levels have been associated with more severe disease [20,39], but whether they independently predict a poorer response to standard non-surgical therapy, after accounting for baseline clinical severity, has not been established. It has been hypothesized that the baseline RANKL/OPG ratio might reflect bone regenerative potential [36]; this has not been tested in studies of regenerative outcomes. Genetic polymorphisms in inflammatory genes (e.g., IL-1A, IL-1B, TNF-α), combined with baseline biomarker levels, have been proposed for identifying hyperinflammatory phenotypes, although findings on IL-1 genotypes have been inconsistent [28,47].

Post-treatment monitoring and early response: Biomarker measurements at early time points after treatment (1–4 weeks) might provide early information on treatment response. In longitudinal studies, salivary IL-1β and IL-6 decreased within the first weeks after non-surgical therapy in patients with favorable clinical responses, whereas persistently elevated levels were observed in some patients with poorer responses [33,39]. Similarly, persistently elevated MMP-8 after therapy has been associated with residual inflammation [14,39]. These observations derive from a small number of studies with limited sample sizes and follow-up, and they have not been translated into validated time points or thresholds; whether early biomarker changes predict long-term stability independently of clinical re-evaluation remains to be established in adequately powered prospective studies.

Salivary markers and implant outcomes: In implant dentistry, it has been proposed that pre-surgical salivary biomarker profiling could identify patients at elevated risk for peri-implantitis or implant failure. Elevated baseline levels of inflammatory cytokines, particularly in patients with a history of periodontitis, may indicate heightened susceptibility to peri-implant disease [13,75]. Genetic polymorphisms in inflammatory genes, such as the IL-1 gene cluster, have been examined in relation to peri-implantitis, with inconsistent results [47]. Integration of salivary biomarkers with clinical risk factors (smoking, diabetes, history of periodontitis), microbiological profiles, and genetic markers is being explored for developing implant risk prediction models [75,76]; however, these models are at an early stage of development, and none has been externally validated.

Non-responder identification: Early identification of non-responders—patients who fail to achieve adequate healing despite standard therapy—is a potential application of monitoring biomarkers. In the available studies, patients with poorer clinical responses tended to show persistently elevated inflammatory biomarkers (IL-1β, IL-6, TNF-α) and tissue destruction markers (MMP-8, MMP-9) at 3–6 months post-treatment [33,39]. If confirmed prospectively, such findings could support earlier consideration of additional interventions, such as adjunctive antimicrobials, surgical therapy, or more frequent maintenance [33]; at present, however, clinical re-evaluation remains the basis for these decisions.

Biomarker trajectories and healing phenotypes: Based on the limited longitudinal data available, we propose—as a hypothesis to be tested—that distinct healing phenotypes may exist. “Rapid responders” might show steep declines in inflammatory biomarkers within 2–4 weeks and near-normal levels by 3 months [33,39]; “slow responders” might show gradual declines over 6–12 months with eventual stability [33]; and “non-responders” might show minimal decline or even increases despite treatment [33,39]. If such phenotypes were confirmed and shown to be associated with long-term outcomes, they could inform the planning of maintenance intervals [10,33].

Predictive multi-marker panels: Single biomarkers have limited discriminatory accuracy, but multi-marker panels combining inflammatory cytokines, tissue destruction markers, and microbial signatures show improved performance [6,25,26]. For example, a panel combining IL-1β, ICTP (bone resorption marker), and *P. gingivalis* DNA distinguished periodontitis from health and gingivitis with AUC values of 0.94 and 0.77 respectively [25]. Another study found that IL-1β and MMP-8 combined could distinguish gingivitis from health with AUC of 0.84 [25]. These multi-marker approaches capture different aspects of disease pathophysiology and provide more robust discrimination than single biomarkers, although external validation of such panels is generally lacking [6,26].

Integration with clinical parameters: Prediction is likely to be improved by integrating salivary biomarkers with traditional clinical parameters and risk factors. Biomarkers provide information about molecular disease activity that complements clinical measures of tissue destruction [6,10]. Risk assessment tools incorporating biomarkers, clinical parameters, genetic factors, and patient characteristics (smoking, diabetes, compliance) may eventually support risk stratification [28,40]. Machine learning algorithms trained on multi-dimensional datasets are being explored for developing such models, but external validation is generally lacking [75,76,77].

## 10. Temporal Healing Dynamics and Monitoring

Understanding the temporal dynamics of salivary biomarkers during healing is essential for interpreting biomarker data and distinguishing normal healing responses from pathological processes [33,39]. Different biomarkers exhibit distinct kinetics, reflecting their roles in sequential phases of inflammation, tissue remodeling, and regeneration [20,33]. The time windows described below are approximate, are derived from a limited number of longitudinal studies with heterogeneous designs [33,39], and should be regarded as indicative rather than as validated reference intervals.

Salivary biomarker trajectories after non-surgical therapy: Non-surgical periodontal therapy (scaling and root planing) produces characteristic temporal biomarker changes. Acute inflammatory cytokines (IL-1β, IL-6, IL-8, TNF-α) have been reported to decline within approximately 1–4 weeks after treatment in successfully treated patients [33,39,49,50]. This rapid decline reflects resolution of acute inflammation and reduction in bacterial load [33]. Tissue destruction markers (MMP-8, MMP-9, elastase) show intermediate-term responses, declining over 4–12 weeks as tissue remodeling progresses and collagen degradation decreases [14,33,39]. Bone remodeling markers (RANKL/OPG ratio) show longer-term changes over 3–6 months, reflecting the slower kinetics of bone healing [20,33]. Microbial markers (*P. gingivalis*, total bacterial load) decline rapidly after mechanical debridement but may show partial rebound if oral hygiene is inadequate [16].

Surgical treatment responses: Surgical periodontal therapy produces more complex biomarker trajectories. Immediately after surgery (days 1–7), transient elevations in inflammatory cytokines and MMPs may occur, reflecting normal wound healing responses including neutrophil infiltration, angiogenesis, and tissue remodeling [33]. This acute post-surgical elevation should not be interpreted as treatment failure but rather as physiological healing [33]. In successfully treated patients, inflammatory biomarkers would be expected to fall below pre-treatment levels within the following weeks, and MMP-8 may remain modestly elevated during the remodeling phase before approaching health-associated levels, plausibly within approximately 3 months [14,33,39]. Persistently elevated biomarkers beyond this period may warrant clinical re-evaluation for inadequate healing or early recurrence; however, these time frames are extrapolated mainly from studies of non-surgical therapy and have not been validated for surgical procedures.

Supportive periodontal care monitoring: During the maintenance phase, salivary biomarkers could support monitoring for early signs of disease recurrence. Stable patients show consistently low biomarker levels at maintenance visits [10,33]. Gradual increases in IL-1β, MMP-8, or other inflammatory markers over successive maintenance visits may indicate subclinical disease reactivation before clinical parameters detect changes [33,39]. Such early warning could prompt intensified treatment (more frequent maintenance, localized antimicrobials) to prevent progression [10,33]. Biomarker-guided maintenance intervals—adjusting visit frequency based on biomarker levels rather than fixed schedules—represent a hypothesis that has not yet been tested in clinical trials [10,40].

Implant placement and peri-implant healing: Following implant placement, salivary biomarkers reflect the healing and osseointegration process. Alkaline phosphatase, a marker of osteoblast activity and bone formation, shows temporal changes related to bone healing around implants [78]. Inflammatory cytokines should decline as initial surgical inflammation resolves and osseointegration progresses [78]. Persistently elevated inflammatory markers during the healing phase may indicate complications such as infection or inadequate osseointegration [78]. Longitudinal biomarker monitoring during implant healing may enable early detection of problems and intervention to prevent implant failure [78].

Temporal patterns in peri-implant therapy: Treatment of peri-implantitis produces biomarker trajectories similar to periodontal therapy but potentially with slower kinetics due to the challenges of implant surface decontamination [45]. As noted in Section 8, salivary sTREM-1 and MMP-8 decreased over 3–6 months after surgical treatment of peri-implantitis in successfully treated patients [45]. Patients showing minimal biomarker decline may require additional treatment or more frequent monitoring [45].

Biomarker stability and reference ranges: Establishing stable biomarker levels in health and defining reference ranges for different disease states is essential for clinical interpretation. Healthy individuals show relatively stable, low levels of inflammatory biomarkers over time, though some intra-individual variability exists due to factors such as oral hygiene, diet, and circadian rhythms [10,28]. Periodontitis patients show higher baseline levels and greater variability [10]. Following successful treatment, biomarker levels should stabilize at values approaching healthy reference ranges, though complete normalization may not always occur [33,49]. Defining patient-specific baselines and monitoring changes from baseline may be more informative than comparing to population reference ranges [10].

## 11. Proteomic, Genomic, and Metabolomic Approaches

**Figure 9:**
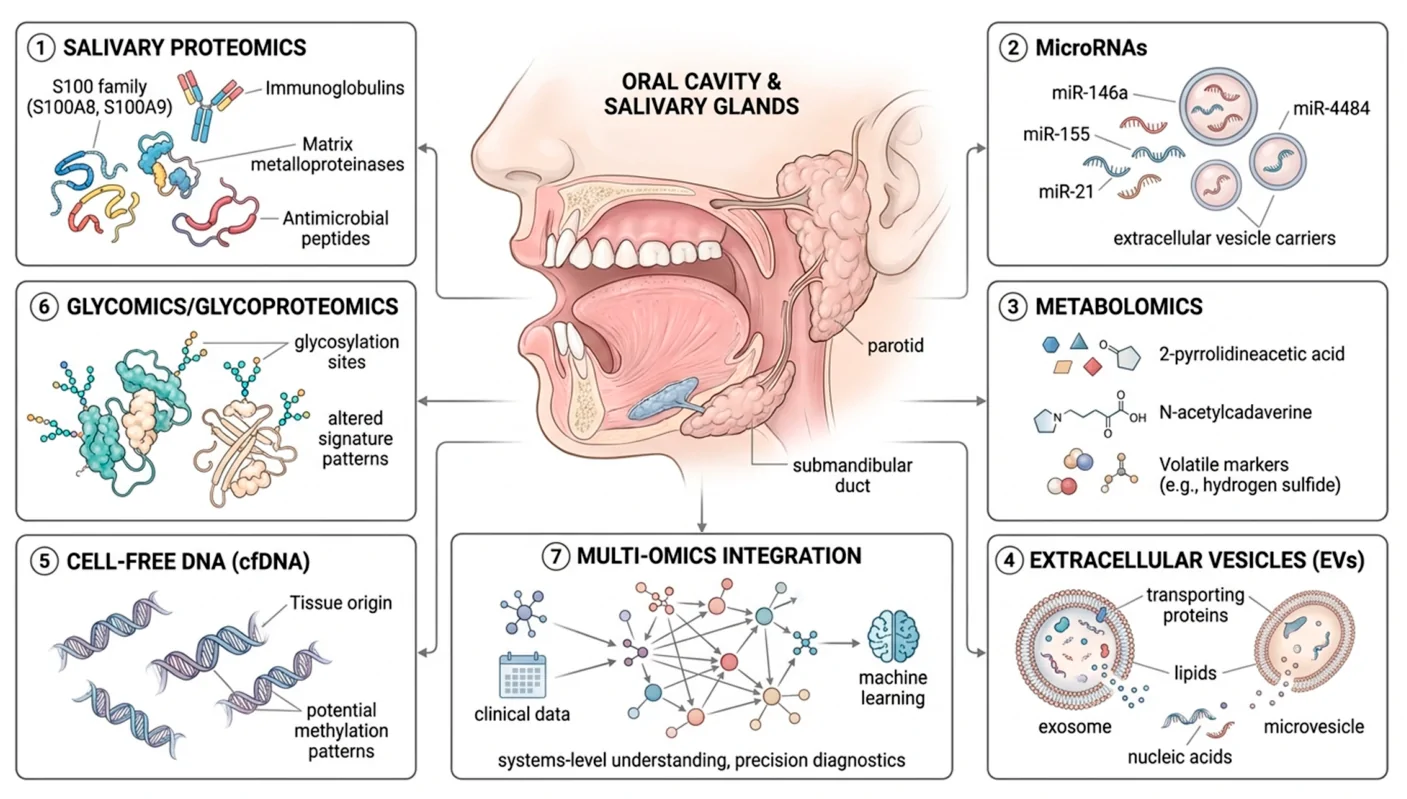
Emerging multi-omics approaches for salivary biomarker discovery and precision diagnostics. Saliva provides access to complementary molecular layers, including the proteome, microRNAs, metabolome, extracellular-vesicle cargo, cell-free DNA, and glycosylation signatures. Integration of these datasets using computational and machine-learning approaches can identify multidimensional biomarker signatures that better reflect host inflammation, microbial activity, tissue destruction, and systemic influences than individual biomarkers, supporting disease stratification, monitoring, and precision oral diagnostics Schematic illustration (Created with FigureLab v2.1).

While targeted measurement of individual biomarkers has dominated periodontal diagnostics research, comprehensive omics-based approaches offer the potential to discover novel biomarkers, identify multi-marker signatures, and gain systems-level understanding of disease pathophysiology [16,19,41,42] (Figure 9).

Relevance to the salivary healing signature: Within the framework proposed in Section 4, omics technologies serve two purposes. First, untargeted proteomic, metabolomic, transcriptomic, and microbiome analyses are discovery tools that may identify new candidate components for each domain of the signature—for example, calprotectin and other neutrophil-derived proteins for the tissue-remodeling domain, microRNAs regulating inflammatory signaling for the inflammatory domain, and taxa or metabolic pathways for the microbial domain. Second, omics profiling before and after therapy can reveal which molecules change in parallel with clinical healing and might therefore serve as monitoring markers. In both cases, omics-derived candidates must subsequently be translated into targeted, quantitative assays and validated in independent cohorts before they can contribute to a clinically usable signature [28,40].

Salivary proteomics panels: Mass spectrometry-based proteomics enables unbiased profiling of hundreds to thousands of proteins in saliva simultaneously [19,41,42]. Multiple proteomic studies have compared salivary protein profiles between periodontitis patients and healthy controls, identifying differentially expressed proteins including S100 family members (S100A8, S100A9, S100A12), immunoglobulins, matrix metalloproteinases, antimicrobial peptides, and numerous other proteins [41,42]. The S100 protein family, particularly S100A8/A9 (calprotectin), has emerged as a consistent finding across multiple proteomic studies and shows strong associations with periodontal disease severity [49]. Proteomic approaches have also identified novel candidate biomarkers not previously investigated in periodontal disease, expanding the repertoire of potential diagnostic markers [41,42]. A recent study using salivary proteomics and intact N-glycopeptide analysis identified specific protein signatures and glycosylation patterns distinguishing periodontitis from health [79]. Proteomic profiling has been applied to monitor treatment response, revealing dynamic changes in salivary protein composition following non-surgical therapy [80]. Multi-protein panels derived from proteomic studies show promise for improving diagnostic accuracy beyond single biomarkers [81].

MicroRNAs (miRNAs): These small non-coding RNAs regulate gene expression and are emerging as stable biomarkers in saliva [16,17,44]. Several miRNAs have been investigated in periodontal disease, with miR-146a, miR-155, and miR-21 showing particular promise [16,17,44]. MiR-146a is involved in regulating inflammatory responses and shows altered expression in periodontitis [44]. MiR-155 promotes inflammatory cytokine production and is upregulated in periodontal disease [44]. MiR-21 regulates cell proliferation, apoptosis, and inflammation and shows differential expression in periodontitis [16]. In peri-implant disease, miR-4484 combined with CXCL14 showed high diagnostic accuracy in a single study for peri-implantitis detection [66]. MicroRNAs are relatively stable in saliva due to protection by extracellular vesicles and protein complexes, making them attractive biomarkers [16]. Panels of multiple miRNAs may provide superior diagnostic performance compared to single miRNAs [16,44].

Metabolomics: Salivary metabolomic profiling using mass spectrometry identifies small molecule metabolites reflecting microbial metabolism, host metabolism, and tissue breakdown [46]. A population-based study using untargeted metabolomics identified 562 salivary metabolites and found that specific metabolites including 2-pyrrolidineacetic acid, butyrylputrescine, N-acetylcadaverine, and N,N,N-trimethyllysine were associated with periodontitis and predicted 5-year tooth loss [46]. These metabolites likely reflect bacterial metabolism (polyamines from amino acid degradation) and tissue breakdown processes [46]. Metabolomic approaches may identify novel biomarkers complementary to protein-based markers and provide insights into disease mechanisms [46]. Volatile metabolites contributing to oral malodor, including hydrogen sulfide and methyl mercaptan, have also been investigated as potential biomarkers [20].

Extracellular vesicles (EVs): Salivary EVs, including exosomes (30–150 nm) and microvesicles (100–1000 nm), carry proteins, lipids, and nucleic acids and serve as intercellular communication vehicles [16]. EVs are secreted by virtually all cell types in periodontal tissues and enter saliva via GCF [16]. Salivary EVs from periodontitis patients show distinct protein and miRNA cargo compared to healthy controls [16]. EVs may serve as stable carriers protecting biomarkers from degradation in the oral environment [16]. EV-based biomarkers are being explored for periodontal diagnostics, and salivary EVs have been investigated for their potential therapeutic applications in tissue regeneration [35].

Cell-free DNA (cfDNA): Circulating cell-free DNA in saliva originates from dying cells in periodontal tissues and may reflect tissue damage and cellular stress [16]. Elevated levels of cfDNA have been detected in periodontitis patients [16]. Bacterial cell-free DNA in saliva provides information about microbial load and composition [16]. Analysis of cfDNA methylation patterns may provide epigenetic biomarkers of disease [82].

Glycomics and glycoproteomics: Protein glycosylation patterns are altered in inflammation and may serve as disease biomarkers. A recent study using intact N-glycopeptide analysis identified specific glycosylation signatures distinguishing periodontitis from health [79]. Glycomic approaches may reveal novel biomarkers and provide insights into inflammatory processes [79].

Multi-omics integration: The most comprehensive understanding of periodontal disease will come from integrating multiple omics layers—proteomics, genomics, transcriptomics, metabolomics, and microbiomics [16,18,40]. Multi-omics approaches can identify molecular networks and pathways dysregulated in disease, reveal interactions between host and microbial factors, and enable systems-level understanding of pathophysiology [40]. Integration of multi-omics data with clinical parameters using machine learning may enable development of diagnostic and prognostic models [40,77]. Such approaches may contribute to more individualized periodontal care, although they currently remain research tools [40].

## 12. Point-of-Care Technologies and Digital Integration

Translation of salivary biomarkers into clinical practice requires point-of-care (POC) technologies that enable rapid, accurate, and cost-effective chairside testing without requiring specialized laboratory equipment or personnel [28,43,44,83]. Recent advances in biosensor technology, microfluidics, and digital health integration are bringing this vision closer to reality [43,44,83].

Relevance to the salivary healing signature: Because the healing signature is defined by change over time rather than by a single measurement, its clinical application would depend on inexpensive, rapid, and reproducible tests that can be repeated at baseline, re-evaluation, and maintenance visits. Point-of-care platforms are therefore a practical prerequisite for serial monitoring. Conversely, the multi-domain nature of the signature implies that single-analyte tests, such as current aMMP-8 lateral-flow devices, capture only one of its components, and that multiplexed platforms with quantitative output would be required to implement it. Table 3 compares the main technologies with respect to these requirements.

Electrochemical biosensors: detect biomarkers through electrochemical reactions that generate measurable electrical signals proportional to biomarker concentration [43,44,83]. These sensors typically use antibodies or aptamers immobilized on electrode surfaces to capture target biomarkers, followed by electrochemical detection [43,44]. Electrochemical biosensors for salivary MMP-8, IL-1β, IL-6, and other periodontal biomarkers have been developed and show promising analytical performance [43,44,83]. Advantages include rapid results (minutes), small sample volumes (microliters), low cost, and potential for miniaturization into handheld devices [43,44]. Challenges include maintaining antibody stability, preventing non-specific binding, and achieving sensitivity comparable to laboratory-based immunoassays [43,44].

Lateral flow immunoassays (LFIAs): These paper-based devices, similar to pregnancy tests, provide simple visual readouts for biomarker detection [43,44]. LFIAs use antibody-conjugated gold nanoparticles or other labels that produce colored lines when target biomarkers are present [43]. Commercial LFIAs for salivary MMP-8 are available and have been validated for periodontal disease screening [56,57,59]. LFIAs offer extreme simplicity and low cost but typically have lower sensitivity than laboratory methods and provide only semi-quantitative results [43,44].

Microfluidic devices (lab-on-a-chip): Microfluidic platforms integrate sample processing, biomarker detection, and data analysis on miniaturized chips [43,44,55,83]. These devices can perform multiple assays simultaneously (multiplexing), require minimal sample volumes, and provide quantitative results [43,44]. Microfluidic devices for salivary biomarker detection have been developed using various detection methods including electrochemical, optical, and fluorescence-based approaches [43,44,55,83]. Lab-on-a-chip technology may enable comprehensive multi-biomarker profiling at the point of care [32,43,44].

Smartphone-based detection: Integration of biosensors with smartphones enables portable, connected diagnostic devices [43,83]. Smartphones can serve as optical detectors (using camera and image analysis), provide user interfaces for test operation, store and transmit data, and connect to cloud-based analysis platforms [43,83]. Smartphone-based colorimetric and fluorescence detection systems for salivary biomarkers have been demonstrated [43,83]. This approach leverages the ubiquity of smartphones to create accessible diagnostic tools [43,83].

AI/ML risk classification: Artificial intelligence and machine learning algorithms can integrate multi-biomarker data with clinical parameters, patient characteristics, and historical data to generate risk estimates [75,76,77]. Machine learning models trained on large datasets of biomarker measurements and clinical outcomes can identify complex patterns and interactions not apparent to human clinicians [77]. AI-driven risk stratification tools are being developed for periodontal disease progression prediction and implant success prediction [75,76,77]. At present, such tools remain at an early stage of development, are typically trained on small, single-center datasets, and require external validation before clinical use [75,76,77].

Commercial POC tests: Several point-of-care tests for salivary biomarkers have achieved commercial availability. The PerioSafe^®^ and ImplantSafe^®^ tests detect active MMP-8 using lateral flow immunoassay technology and are marketed for periodontal and peri-implant disease screening [56,57,59]. These tests provide results in 5 min and have been evaluated in clinical studies [56,57,59]. Other commercial tests under development target multiple biomarkers simultaneously [43,44].

Digital integration and teledentistry: Point-of-care biomarker testing integrated with digital health platforms enables remote monitoring and teledentistry applications [17,83]. Patients could perform home-based saliva testing using simple devices, with results transmitted to dental providers for remote assessment [17,83]. This approach could enable more frequent monitoring, early detection of disease recurrence, and reduced need for in-office visits [17,83]. Integration with electronic health records and clinical decision support systems could facilitate biomarker-guided treatment planning [40].

Challenges for POC translation: Despite technological advances, several challenges remain for widespread POC adoption. Analytical performance (sensitivity, specificity, reproducibility) of POC devices must match or approach laboratory-based methods [28,43]. Standardization of devices, protocols, and interpretation criteria is essential for reliable results across different settings [28]. Regulatory approval (FDA clearance or equivalent) is required for clinical use [28]. Cost-effectiveness must be demonstrated to justify adoption [28]. Clinician education and workflow integration are necessary for successful implementation [28]. Addressing these challenges will be critical for realizing the potential of POC salivary diagnostics [28,43].

## 13. Clinical Translation, Limitations, and Research Priorities

Despite consistent evidence of diagnostic associations, translation of salivary biomarkers into routine clinical practice has been slow [6,10,28]. Multiple barriers must be overcome before salivary diagnostics can contribute to periodontal care [28].

Standardization issues represent a major challenge. Lack of standardized protocols for saliva collection, processing, storage, and analysis contributes to variability across studies and limits comparability of results [10,28]. Different collection methods (unstimulated vs. stimulated saliva, whole saliva vs. GCF), collection times, pre-collection restrictions, and storage conditions all affect biomarker measurements [10,28]. Establishing consensus protocols and standard operating procedures is essential for reliable clinical implementation [28]. Pre-analytical variables including circadian rhythms, recent food intake, smoking, oral hygiene, and medications must be controlled or accounted for [10,28] (Section 3). Development of collection devices that minimize variability and ensure sample quality is needed [28].

Sensitivity and specificity trade-offs affect clinical utility. While many biomarkers show statistically significant differences between health and disease, the degree of overlap between groups limits diagnostic accuracy [26,27]. Single biomarkers typically achieve sensitivity and specificity in the 70–85% range, which may be insufficient for definitive diagnosis [26,27]. Multi-marker panels improve performance but add complexity and cost [6,26]. Defining optimal cutoff values that balance sensitivity (detecting disease) and specificity (avoiding false positives) requires large validation studies [26,27]. The clinical context (screening vs. diagnosis vs. monitoring) influences the optimal sensitivity/specificity balance [28].

Regulatory approval is required for clinical use of diagnostic tests. In the United States, the FDA regulates in vitro diagnostic devices, requiring demonstration of analytical validity (accuracy, precision, reproducibility), clinical validity (sensitivity, specificity for intended use), and clinical utility (impact on patient outcomes) [28]. The regulatory pathway depends on risk classification and intended use [28]. Few salivary biomarker tests have achieved FDA clearance or equivalent regulatory approval in other jurisdictions [28]. The regulatory process is time-consuming and expensive, creating barriers for commercialization [28].

Integration into clinical workflows requires practical considerations. Chairside tests must be rapid (ideally <10 min), simple to perform without specialized training, and provide clear, actionable results [28]. The test must fit into existing appointment structures without significantly extending visit times [28]. Results must be interpretable by general dentists, not just specialists [28]. Integration with practice management software and electronic health records facilitates documentation and longitudinal tracking [40]. Clinical decision support tools that integrate biomarker results with clinical parameters and provide treatment recommendations may enhance utility [40].

Cost-effectiveness must be demonstrated for widespread adoption. The cost of biomarker testing must be justified by improved outcomes, reduced treatment costs, or other benefits [28]. Cost-effectiveness analyses should consider direct costs (test materials, equipment, labor) and indirect costs (time, training) as well as benefits including earlier disease detection, reduced need for invasive procedures, and improved long-term outcomes [28]. Reimbursement by insurance or health systems is critical for sustainability [28]. Currently, most salivary biomarker tests are not covered by insurance, limiting adoption [28].

Clinician education and acceptance are essential. Dentists must understand the rationale for biomarker testing, interpretation of results, and integration with clinical decision-making [28]. Educational programs, continuing education courses, and clinical guidelines are needed [28]. Demonstrating clinical utility through well-designed studies showing that biomarker-guided care improves outcomes compared to standard care is critical for acceptance [6,28]. Overcoming skepticism and inertia in clinical practice requires compelling evidence and effective communication [28].

Potential opportunities for clinical impact: If these challenges are addressed, salivary biomarkers could contribute to periodontal care in several ways [6,10,17]: earlier detection of disease activity [17,32,84]; risk stratification [10,40]; monitoring of treatment response [10,33,39]; adjustment of maintenance intervals [10,40]; provision of objective molecular data that complement clinical assessment [6,10]; and patient education and motivation [28]. Each of these potential applications requires confirmation in prospective studies [6,10,28].

Limitations of the current evidence: Several limitations of the evidence base should be emphasized. Most studies are cross-sectional or case–control comparisons of well-defined disease and health groups, a design that tends to inflate estimates of diagnostic accuracy; sample sizes are frequently small; case definitions vary, and many studies do not apply the 2017 classification; assays, units, and normalization methods differ, precluding the derivation of common thresholds; confounders such as smoking, diabetes, and medication use are inconsistently reported; follow-up after therapy is generally short; and external validation of multimarker panels or prediction models is rare. In addition, studies originate from a limited number of populations, which restricts generalizability, and publication bias toward positive associations is likely. This review also has limitations inherent to its narrative design, including the absence of a formal risk-of-bias assessment and quantitative synthesis, and the possibility that relevant studies were missed.

Research priorities: To move the salivary healing signature from concept to clinical tool, future research should (i) adopt standardized and fully reported protocols for saliva collection, processing, and analysis; (ii) conduct adequately powered prospective cohort studies in diverse populations, with repeated biomarker measurements and clinically meaningful endpoints such as disease progression, tooth loss, or implant loss; (iii) prespecify candidate panels and algorithms and validate them externally; (iv) report discrimination, calibration, and the added value of biomarkers beyond established clinical predictors; (v) establish saliva biobanks that allow new candidates to be evaluated; and (vi) ultimately test, in randomized trials, whether biomarker-informed management improves patient outcomes compared with standard care [6,28,40,85].

## 14. Conclusions

Salivary biomarkers—including inflammatory cytokines, matrix metalloproteinases, bone-remodeling markers, microbial signatures, and emerging omics-based candidates—are consistently associated with periodontal and peri-implant disease, and several decrease after successful therapy. Multimarker panels generally discriminate periodontitis from health better than single markers, although current accuracy estimates derive largely from case–control studies. Peri-implant diseases share most inflammatory pathways with periodontitis, and no biomarker profile specific to peri-implantitis has been established.

The salivary healing signature proposed here is a conceptual framework that integrates these biomarker domains with their expected changes over time; it is not a validated clinical test. Its potential role is to complement, not replace, clinical and radiographic assessment, particularly for patient-level longitudinal monitoring, whereas site-specific information continues to require clinical examination and crevicular fluid analysis. Translation will require standardized pre-analytical and analytical protocols, systematic control of confounders, prospective and externally validated studies that distinguish diagnostic, monitoring, prognostic, and predictive applications, and evidence that biomarker-informed care improves patient outcomes.

## Figures and Tables

**Table 1:** Biomarker categories according to intended use (adapted from the BEST framework [21]) and current status of salivary evidence in periodontal and peri-implant diseases.

Category	Clinical Question	Study Design Required	Current Status of Salivary Evidence
Diagnostic	Is periodontitis or peri-implantitis present?	Cross-sectional diagnostic-accuracy studies in the intended-use population against a clinical reference standard	Most abundant; predominantly case–control designs, which are likely to overestimate accuracy [26,27]
Monitoring	Is the disease responding to therapy or remaining stable?	Longitudinal studies with repeated biomarker and clinical measurements	Moderate; mostly short-term pre-/post-treatment comparisons in small samples [33,39,45]
Prognostic	Who is likely to progress, relapse, or lose teeth/implants?	Prospective cohorts with defined clinical endpoints and adjustment for established clinical predictors	Limited; few prospective studies (e.g., salivary metabolites and 5-year tooth loss) [46]
Predictive	Who will benefit more from a specific treatment than from an alternative?	Randomized or controlled comparative studies testing a biomarker-by-treatment interaction	Not established for any salivary biomarker
Susceptibility/risk	Who is at risk of developing disease while currently healthy?	Prospective cohorts of individuals without disease	Very limited; genetic markers (e.g., IL-1 polymorphisms) inconsistent [47]

BEST, Biomarkers, EndpointS, and other Tools.

**Table 2:** Principal salivary biomarkers in periodontal and peri-implant diseases: biological domain and source, main findings, predominant type of evidence, and key limitations.

Biomarker	Domain/Main Source	Principal Salivary Findings	Predominant Evidence	Key Limitations	Ref.
IL-1β	Inflammatory; macrophages, monocytes, epithelium (via GCF)	Higher in periodontitis; correlates with PD, CAL, and BOP; decreases after therapy	Diagnostic (systematic reviews); monitoring (few studies)	Also elevated in gingivitis; influenced by smoking and systemic inflammation; no agreed cut-off	[20,26,27,39,50,51]
IL-6	Inflammatory; macrophages, fibroblasts, endothelium	Elevated; correlates with severity; complementary in panels	Diagnostic	Low concentrations; systemic contributions (e.g., obesity, diabetes)	[25,32,52,53]
IL-8/CXCL8	Inflammatory chemokine; epithelium, macrophages	Elevated; decreases after therapy	Diagnostic; limited monitoring	Inconsistent findings; elevated in other oral inflammation	[50,52]
IL-17	Inflammatory; Th17 cells	Elevated in periodontitis; combined with RANKL/OPG discriminates health and disease	Diagnostic (small studies)	Few studies; heterogeneous assays; peri-implant data limited	[34,36,54]
TNF-α	Inflammatory; macrophages	Elevated in some studies; included in panels	Diagnostic	Frequently near detection limits; inconsistent	[20,52,55]
MMP-8 (total/active)	Tissue remodeling; neutrophils	Elevated; correlates with PD, CAL, and bone loss; decreases after periodontal and peri-implant therapy; aMMP-8 chairside tests available	Diagnostic (systematic reviews); monitoring; limited prognostic	Total and active forms and assays not interchangeable; affected by smoking	[14,26,39,45,56,57,58,59]
MMP-9/TIMP-1	Tissue remodeling; neutrophils, macrophages	MMP-9 elevated; altered MMP/TIMP balance	Diagnostic (limited)	Few salivary validation studies	[35]
Calprotectin (S100A8/A9)	Neutrophil cytosolic protein	Consistently increased in proteomic studies; correlates with severity	Discovery/diagnostic	Mainly proteomic discovery; few targeted validations	[41,42,49]
RANKL/OPG ratio	Bone remodeling; osteoblasts, T cells	Higher RANKL/OPG in periodontitis and peri-implantitis	Diagnostic (small studies)	Inconsistent, particularly for OPG	[36,37,60]
ICTP/CTX, osteocalcin	Bone turnover	Detected in saliva; ICTP included in multimarker panels	Diagnostic (limited)	Low salivary concentrations; sparse data	[20,25]
*P. gingivalis*, *T. forsythia*, *T. denticola* DNA	Microbial	Higher detection and load in periodontitis; decrease after therapy	Diagnostic; limited monitoring	Detected in some healthy individuals; DNA does not indicate viability or activity	[25,38]
Salivary microbiome profiles	Microbial community	Discriminate periodontitis and peri-implantitis from health in some studies	Diagnostic (exploratory)	Methodological heterogeneity; inconsistent diversity findings	[16,61,62,63]
Oxidative stress markers (MDA, TAC, 8-OHdG)	Oxidative damage/antioxidant capacity	MDA higher and TAC lower in peri-implant disease	Diagnostic (small studies; one meta-analysis)	Non-specific; influenced by smoking and systemic disease	[64,65]
CXCL14 + miR-4484	Chemokine/microRNA	Discriminated peri-implantitis from health in one study	Exploratory diagnostic	Single study; replication required	[66]
Multimarker panels (e.g., IL-1β + ICTP + *P. gingivalis*)	Combined domains	AUC up to 0.94 for periodontitis versus health	Diagnostic	Mostly case–control; risk of overfitting; external validation lacking	[25,26,27]

8-OHdG, 8-hydroxydeoxyguanosine; AUC, area under the receiver operating characteristic curve; BOP, bleeding on probing; CAL, clinical attachment loss; CTX, C-terminal telopeptide of type I collagen; GCF, gingival crevicular fluid; ICTP, carboxy-terminal telopeptide of type I collagen; IL, interleukin; MDA, malondialdehyde; MMP, matrix metalloproteinase; OPG, osteoprotegerin; PD, probing depth; RANKL, receptor activator of nuclear factor-κB ligand; TAC, total antioxidant capacity; TIMP, tissue inhibitor of metalloproteinases; TNF, tumor necrosis factor.

**Table 3:** Comparison of point-of-care and reference technologies for salivary biomarker detection in periodontal and peri-implant diseases.

Technology	Detection Principle	Example Targets	Output/Multiplexing	Time to Result	Development Stage	Main Advantages	Main Limitations
Lateral flow immunoassay [43,56,57,59]	Antibody capture with colorimetric (e.g., gold-nanoparticle) readout; optional digital reader	aMMP-8	Qualitative/semi-quantitative (quantitative with reader); single analyte	5–10 min	Commercially available (e.g., PerioSafe^®^, ImplantSafe^®^); clinical evaluation studies	Simple; low cost; chairside	Single analyte; threshold-based; accuracy varies with population and case definition
Electrochemical biosensor [43,44,83]	Antibody/aptamer recognition on electrode; current or impedance change	MMP-8, IL-1β, IL-6	Quantitative; limited multiplexing	Minutes	Laboratory prototypes; few clinical validations	High sensitivity; small sample volume; miniaturizable	Biofouling and non-specific binding in saliva; reagent stability; calibration
Microfluidic lab-on-a-chip [43,44,55,83]	Integrated sample processing with optical, electrochemical, or fluorescence detection	Cytokine and MMP panels	Quantitative; multiplex	10–60 min	Prototypes; small feasibility studies	Multiplexing; automation; minimal sample	Manufacturing complexity and cost; salivary viscosity; regulatory pathway
Smartphone-based reader [43,83]	Smartphone camera/optics with app-based analysis of colorimetric or fluorescence signals	MMP-8; colorimetric assays	Semi-quantitative to quantitative; data transmission	Minutes	Proof-of-concept	Accessibility; connectivity; data storage	Device-to-device variability; lighting conditions; data security; validation lacking
Laboratory multiplex immunoassay (reference) [6]	Bead-based or electrochemiluminescence immunoassay	10–40 cytokines and growth factors	Quantitative; high multiplex	Hours to days	Established research platforms	Reference method for discovery and validation	Not chairside; cost; inter-platform differences

aMMP-8, active matrix metalloproteinase-8; IL, interleukin; MMP, matrix metalloproteinase.

## Data Availability

No new data were created or analyzed in this study. Data sharing is not applicable to this article.

## References

[1] Cafferata E.A. (2026). Periodontal Health and Disease. Sci. Rep..

[2] Kinane D.F., Bıyıkoğlu B. (2026). Diagnostic Biomarkers of Periodontitis and Peri-Implantitis: The Past, the Present, the Future. J. Periodontal Res..

[3] Pliavga V., Peceliunaite G., Daugela P., Leketas M., Gervickas A., Juodzbalys G. (2023). Peri-Implantitis Diagnosis and Prognosis Using Biomarkers: A Systematic Literature Review. Int. J. Oral Maxillofac. Implant..

[4] Derks J., Tomasi C. (2015). Peri-implant health and disease. A systematic review of current epidemiology. J. Clin. Periodontol..

[5] Genco R.J., Sanz M. (2020). Clinical and public health implications of periodontal and systemic diseases: An overview. Periodontol. 2000.

[6] Ebersole J.L., Hasturk H., Huber M., Gellibolian R., Markaryan A., Zhang X.D., Miller C.S. (2024). Realizing the clinical utility of saliva for monitoring oral diseases. Periodontol. 2000.

[7] Tonetti M.S., Greenwell H., Kornman K.S. (2018). Staging and grading of periodontitis: Framework and proposal of a new classification and case definition. J. Periodontol..

[8] Papapanou P.N., Sanz M., Buduneli N., Dietrich T., Feres M., Fine D.H., Flemmig T.F., Garcia R., Giannobile W.V., Graziani F. (2018). Periodontitis: Consensus report of workgroup 2 of the 2017 World Workshop on the Classification of Periodontal and Peri-Implant Diseases and Conditions. J. Periodontol..

[9] Jabeen N., Ghafoor S. (2019). Salivary Biomarkers of Chronic Periodontitis. J. Pak. Dent. Assoc..

[10] Korte D.L., Kinney J. (2015). Personalized Medicine: An Update of Salivary Biomarkers for Periodontal Diseases. Periodontol. 2000.

[11] Khalaf S.J., Hamad M.S., Sarhat E.R. (2023). Salivary Biomarkers in Periodontal Diseases: A Review. Tikrit J. Dent. Sci..

[12] Alnasser M. (2023). Salivary Biomarkers in Periodontitis: A Scoping Review. Cureus.

[13] Haririan H., Andrukhov O., Laky M., Rausch-Fan X. (2021). Saliva as a Source of Biomarkers for Periodontitis and Peri-Implantitis. Front. Dent. Med..

[14] Ramenzoni L.L., Hofer D., Solderer A., Wiedemeier D., Attin T., Schmidlin P.R. (2021). Origin of MMP-8 and Lactoferrin Levels From Gingival Crevicular Fluid, Salivary Glands and Whole Saliva. BMC Oral Health.

[15] Avelar F.M., da Silva T.A., Sabino-Silva R., Lanza C.R.M., Azevedo V.A.d.C. (2025). Salivary Biomarkers for Screening and Monitoring of Oral Diseases.

[16] Radu C.M., Radu C.C., Zaha D.C. (2025). Salivary and Microbiome Biomarkers in Periodontitis: Advances in Diagnosis and Therapy. Medicina.

[17] Alavi S.E., Sharma L.A., Sharma A., Shahmabadi H.E. (2025). Salivary Biomarkers in Periodontal Disease: Revolutionizing Early Detection and Precision Dentistry. Mol. Diagn. Ther..

[18] Bagde H. (2025). Emerging Biomarkers in Periodontal Disease: Diagnostic and Prognostic Implications. RJAMS.

[19] Rakhewar P., Gaikwad A., Panpatil S., Chacko L., Patil S., H M.S. (2023). Role of Proteomic Biomarkers in Periodontal Diseases—A Review. Sch. J. Dent. Sci..

[20] Shi F., Liu W., Yao Y., Zhang Q., Chen Z., Xian Y., Sujanamulk B. (2023). Predictive Salivary Biomarkers for Early Diagnosis of Periodontal Diseases. Turk. Biyokim. Dergisi..

[21] FDA-NIH Biomarker Working Group (2016). BEST (Biomarkers, EndpointS, and other Tools) Resource. https://www.ncbi.nlm.nih.gov/books/NBK326791/.

[22] Melguizo-Rodríguez L., Costela-Ruiz V.J., Manzano-Moreno F.J., Ruiz C., Illescas-Montes R. (2020). Salivary Biomarkers and Their Application in the Diagnosis and Monitoring of the Most Common Oral Pathologies. Int. J. Mol. Sci..

[23] Buzalaf M.A.R., Ortiz A.d.C., Carvalho T.S., Fideles S.O.M., Araújo T.T., Moraes S.M., Buzalaf N.R., Reis F.N. (2020). Saliva as a Diagnostic Tool for Dental Caries, Periodontal Disease and Cancer. Expert Rev. Mol. Diagn..

[24] Li Y., Ou Y., Fan K., Liu G. (2024). Salivary Diagnostics: Opportunities and Challenges. Theranostics.

[25] Zhang Y., Kang N., Xue F., Qiao J., Duan J., Chen F., Cai Y. (2021). Evaluation of Salivary Biomarkers for the Diagnosis of Periodontitis. BMC Oral Health.

[26] Arias-Bujanda N., Regueira-Iglesias A., Balsa-Castro C., Nibali L., Donos N., Tomás I. (2020). Accuracy of Single Molecular Biomarkers in Saliva for the Diagnosis of Periodontitis: A Systematic Review and Meta-Analysis. J. Clin. Periodontol..

[27] Kc S., Wang X.Z., Gallagher J.E. (2020). Diagnostic Sensitivity and Specificity of Host-Derived Salivary Biomarkers in Periodontal Disease Amongst Adults: Systematic Review. J. Clin. Periodontol..

[28] Gursoy U.K., Kantarci A. (2022). Molecular Biomarker Research in Periodontology: A Roadmap for Translation of Science to Clinical Assay Validation. J. Clin. Periodontol..

[29] Noh J.-W., Jang J.-H., Yoon H.-S., Kim K.-B., Heo M.-H., Jang H.-E., Kim Y.-J., Lee Y. (2022). Evaluation of Salivary Biomarkers of Periodontal Disease Based on Smoking Status: A Systematic Review. Int. J. Environ. Res. Public Health.

[30] Dhulipalla R., Vudathaneni V.K.P., Bodduru R., Nadella S.B., Kondapaneni A., Boyapati R. (2025). Assessment of Salivary Biomarkers as Predictors of Periodontal Disease Severity in Smokers and Nonsmokers. J. Pharm. Bioallied Sci..

[31] Miller C.S., Ding X., Dawson D.R., Ebersole J.L. (2021). Salivary biomarkers for discriminating periodontitis in the presence of diabetes. J. Clin. Periodontol..

[32] Cafiero C., Spagnuolo G., Marenzi G., Martuscelli R., Colamaio M., Leuci S. (2021). Predictive Periodontitis: The Most Promising Salivary Biomarkers for Early Diagnosis. J. Clin. Med..

[33] Teles F.R.F., Chandrasekaran G., Martin L., Patel M., Kallan M.J., Furquim C., Hamza T., Cucchiara A.J., Kantarci A., Urquhart O. (2024). Salivary and Serum Inflammatory Biomarkers During Periodontitis Progression and After Treatment. J. Clin. Periodontol..

[34] Mohammed M.A., Abbas R.F. (2023). IL-17 and IL-10 as Potential Biomarkers for Diagnosing Periodontitis Patients: A Review. Diyala J. Med..

[35] Thomas J.T., Joseph B., Waltimo T., Anil S. (2024). Matrix Metalloproteinases (MMPs). Periodontium: Is It a Boon or a Bane? Dentistry.

[36] Abdullameer M.A., Abdulkareem A.A. (2023). Diagnostic Potential of Salivary Interleukin-17, RANKL, and OPG to Differentiate Between Periodontal Health and Disease. Health Sci. Rep..

[37] Ali I.H., Salman S.A. (2025). Potential of salivary IL-36γ, IL-38, and RANKL in differentiating periodontal health from periodontitis. Dent. 3000.

[38] Sha A.M., Abdulbaqi H.R., Bin Qasim S.S. (2024). Microbial and Inflammatory Salivary Biomarkers of Periodontal Diseases. Kurd. J. Appl. Res..

[39] Sachelarie L., Stefanescu C.L., Murineanu R.M., Grigorian M., Zaharia A., Scrobota I., Hurjui L.L. (2025). Role of Salivary Biomarkers IL-1β and MMP-8 in Early Detection and Staging of Periodontal Disease. Medicina.

[40] Bostanci N., Belibasakis G.N. (2023). Precision Periodontal Care: From Omics Discoveries to Chairside Diagnostics. Clin. Oral Investig..

[41] Hu H.L., Leung W.K. (2023). Mass Spectrometry-Based Proteomics for Discovering Salivary Biomarkers in Periodontitis: A Systematic Review. Int. J. Mol. Sci..

[42] Corana M., Baima G., Iaderosa G., Franco F., Zhang J., Berta G.N., Romano F., Aimetti M. (2024). Salivary Proteomics for Detecting Novel Biomarkers of Periodontitis: A Systematic Review. J. Periodontal Res..

[43] Griffith A., Chande C., Kulkarni S., Morel J., Cheng Y.-H., Shimizu E., Cugini C., Basuray S., Kumar V. (2024). Point-of-Care Diagnostic Devices for Periodontitis—Current Trends and Urgent Need. Sens. Diagn..

[44] Steigmann L., Maekawa S., Sima C., Travan S., Wang C.-W., Giannobile W.V. (2020). Biosensor and Lab-on-a-Chip Biomarker-Identifying Technologies for Oral and Periodontal Diseases. Front. Pharmacol..

[45] Neves G.S.T., Elangovan G., Teixeira M.K.S., de Mello-Neto J.M., Tadakamadla S.K., Lourenço E.J.V., Telles D.M., Figueredo C.M. (2022). Peri-Implant Surgical Treatment Downregulates sTREM-1 and MMP-8 in Patients with Peri-Implantitis. Int. J. Environ. Res. Public Health.

[46] Andörfer L., Holtfreter B., Weiss S., Matthes R., Pitchika V., Schmidt C.O., Samietz S., Kastenmüller G., Nauck M., Völker U. (2021). Salivary Metabolites Associated with a 5-Year Tooth Loss Identified in a Population-Based Setting. BMC Med..

[47] Santostasi N., Gerardi D., Rinaldi F., Bernardi S., Bianchi I., Pinchi V., Piattelli M., Varvara G. (2024). Relationship Between Interleukin-1 (IL-1) Genetic Polymorphism and Peri-Implantitis: Systematic Literature Review and Meta-Analysis. Eur. Rev. Med. Pharmacol. Sci..

[48] Güncü G.N., Yilmaz D., Könönen E., Gürsoy U.K. (2015). Salivary Antimicrobial Peptides in Early Detection of Periodontitis. Front. Cell Infect. Microbiol..

[49] Sánchez-Medrano A.G., Martinez-Martinez R.E., Soria-Guerra R., Portales-Perez D., Bach H., Martinez-Gutierrez F. (2023). A Systematic Review of the Protein Composition of Whole Saliva in Subjects with Healthy Periodontium Compared with Chronic Periodontitis. PLoS ONE.

[50] Bertoldi C., Nasi M., Salvatori R., Pinti M., Montagna S., Tonetti M., Generali L., Bellei E., Zaffe D., Selleri V. (2025). Salivary Biomarker Analysis to Distinguish Between Health and Periodontitis Status: A Preliminary Study. Dent. J..

[51] Bolyarova-Konova T., Petkova S., Mihaylova H., Velikova T., Ivanova-Todorova E., Tumangelova-Yuzeir K., Todorova D. (2020). Concentrations of Interleukin-1β in Gingival Crevicular Fluid and Saliva—A Potential Diagnostic Biomarker of Periodontal Diseases. Folia Medica.

[52] Majeed Z.N., Jassim S.D., Kamil Z.H. (2023). Assessment of Salivary and GCF IL-6, IL-8, IL-12, and IL-17 Levels in Healthy Subjects and Patients with Periodontal Disease. J. Oral Maxillofac. Pathol..

[53] Salim A., Angelova S., Roussev B., Sokrateva T., Kiselova-Kaneva Y., Peev S., Ivanova D. (2023). Salivary IL-6, IL-1β, and C-Reactive Protein as a Diagnostic Tool for Plaque-Induced Gingivitis in Children. Appl. Sci..

[54] Vahabi S., Yadegari Z., Pournaghi S. (2020). The Comparison of the Salivary Concentration of Interleukin-17 and Interleukin-18 in Patients with Chronic Periodontitis. Dent. Res. J..

[55] Haji Mohammadi M., Mulder S., Khashayar P., Kalbasi A., Azimzadeh M., Aref A.R. (2021). Saliva Lab-on-a-Chip Biosensors: Recent Novel Ideas and Applications in Disease Detection. Microchem. J..

[56] Gul S.S., Abdulkareem A.A., Sha A.M., Rawlinson A. (2020). Diagnostic Accuracy of Oral Fluids Biomarker Profile to Determine the Current and Future Status of Periodontal and Peri-Implant Diseases. Diagnostics.

[57] Lähteenmäki H., Pätilä T., Pärnänen P., Räisänen I., Tervahartiala T., Gupta S., Sorsa T. (2023). aMMP-8 Point-of-Care—Diagnostic Methods and Treatment Modalities in Periodontitis and Peri-Implantitis. Expert Opin. Ther. Targets.

[58] Räisänen I.T., Aji N.R.A.S., Sakellari D., Grigoriadis A., Rantala I., Pätilä T., Heikkilä P., Gupta S., Sorsa T. (2023). Active Matrix Metalloproteinase-8 (aMMP-8) Versus Total MMP-8 in Periodontal and Peri-Implant Disease Point-of-Care Diagnostics. Biomedicines.

[59] Lähteenmäki H., Umeizudike K.A., Heikkinen A.M., Räisänen I.T., Rathnayake N., Johannsen G., Tervahartiala T., Nwhator S.O., Sorsa T. (2020). aMMP-8 Point-of-Care/Chairside Oral Fluid Technology as a Rapid Non-Invasive Tool for Periodontitis and Peri-Implantitis Screening. Diagnostics.

[60] Gomes A.M., Douglas-De-Oliveira D.W., Ferreira S.D., da Silva T.A., Cota L.O.M., Costa F.O. (2019). Periodontal Disease, Peri-Implant Disease and Levels of Salivary Biomarkers IL-1β, IL-10, RANK, OPG, MMP-2, TGF-β and TNF-α: Follow-Up Over 5 Years. Braz. Oral Res..

[61] Pallos D., Sousa V., Feres M., Retamal-Valdes B., Chen T., Curtis M., Boaventura R.M., Tanaka M.H., Salomão G.V.d.S., Zanella L. (2022). Salivary Microbial Dysbiosis Is Associated With Peri-Implantitis: A Case-Control Study in a Brazilian Population. Front. Cell. Infect. Microbiol..

[62] Bessa L.J., Egas C., Pires C., Proença L., Mascarenhas P., Pais R.J., Barroso H., Machado V., Botelho J., Alcoforado G. (2025). Linking Peri-Implantitis to Microbiome Changes in Affected Implants, Healthy Implants, and Saliva: A Cross-Sectional Pilot Study. Front. Cell. Infect. Microbiol..

[63] Belstrøm D. (2020). The salivary microbiota in health and disease. J. Oral Microbiol..

[64] Popa P.Ș., Popa G.V., Earar K., Popa-Cazacu C.E., Matei M.N. (2025). Salivary Oxidative Stress Biomarkers in Peri-Implant Disease: A Systematic Review and Meta-Analysis. Int. J. Mol. Sci..

[65] Karasu Y.Ö., Maden O., Çanakçı C.F. (2024). Oxidative damage biomarkers and antioxidant enzymes in saliva of patients with peri-implant diseases. Int. J. Implant Dent..

[66] Urvasizoglu G., Kilic A., Capik O., Gundogdu M., Karatas O.F. (2023). CXCL14 and miR-4484 serves as potential salivary biomarkers for early detection of peri-implantitis. Odontology.

[67] Abusleme L., Dupuy A.K., Dutzan N., Silva N., A Burleson J., Strausbaugh L.D., Gamonal J., I Diaz P. (2013). The subgingival microbiome in health and periodontitis and its relationship with community biomass and inflammation. ISME J..

[68] Berglundh T., Armitage G., Araujo M.G., Avila-Ortiz G., Blanco J., Camargo P.M., Chen S., Cochran D., Derks J., Figuero E. (2018). Peri-implant diseases and conditions: Consensus report of workgroup 4 of the 2017 World Workshop on the Classification of Periodontal and Peri-Implant Diseases and Conditions. J. Clin. Periodontol..

[69] Mattheos N. (2023). Oral Fluid Biomarkers for Peri-Implantitis: A Scoping Review. Int. Dent. J..

[70] Nayak R., Eweis S.H.A.M., Gupta R.K., Rathore R., Tripathi P., Dahiya V. (2025). Assessment of Salivary Biomarkers in Early Peri-Implantitis Detection. Bioinformation.

[71] Carcuac O., Berglundh T. (2014). Composition of human peri-implantitis and periodontitis lesions. J. Dent. Res..

[72] Titusson C., Jansson L., Modin C., Lundmark A., Eriksson K., Adler L., Yucel-Lindberg T. (2025). Salivary Inflammatory Mediator Profiles in Periodontal and Peri-Implant Health and Disease: A Cross-Sectional Study. Clin. Implant Dent. Relat. Res..

[73] Algohar A., Alqerban A. (2020). Levels of procalcitonin in saliva and peri-implant crevicular fluid in patients with peri-implant diseases and health. Arch. Oral Biol..

[74] Miranda B.P., Miranda H.P., Carreiro R.P.F., Schnurr E. (2021). Salivary Markers as Diagnostic Tool for Dental Caries, Periodontal Disease and Peri-implantitis: A Literature Review. J. Clin. Diagn. Res..

[75] Fragkioudakis I., Batas L., Vouros I., Sakellari D.A. (2025). A Personalized, Biomarker-Based Risk Assessment Model for Peri-Implantitis: Integration of Clinical, Molecular, and Microbial Predictors. Preprints.

[76] Bornes R., Montero J., Rosa N., Fonseca P., Correia A. (2026). Development and Design of Implant Success Prediction Tool: AI Risk Assessment for Peri-Implant Disease Prevention. Int. J. Implant Dent..

[77] Furquim C.P., Caruth L., Chandrasekaran G., Cucchiara A., Kallan M.J., Martin L., Feres M., Bittinger K., Divaris K., Glessner J. (2025). Developing Predictive Models for Periodontitis Progression Using Artificial Intelligence: A Longitudinal Cohort Study. J. Clin. Periodontol..

[78] Hekmat B., Amani M., Aghmasheh F., Hasani Z.M. (2025). Salivary Alkaline Phosphatase Levels and Alveolar Bone Crest Changes in Immediate vs. Delayed Implant Placement. J. Adv. Periodontol. Implant Dent..

[79] Zhao Z., Sun X., Cao L., Zhu C., He K., Hu X., Liu C., Feng Q., Qin Y. (2023). Salivary Proteome and Intact N-Glycopeptides Analysis Reveal Specific Signatures in Periodontitis. J. Proteome Res..

[80] Silbereisen A., Bao K., Wolski W., Nanni P., Kunz L., Afacan B., Emingil G., Bostanci N. (2025). Probing the Salivary Proteome for Prognostic Biomarkers in Response to Non-Surgical Periodontal Therapy. J. Clin. Periodontol..

[81] Grant M.M., Taylor J.J., Jaedicke K., Creese A., Gowland C., Burke B., Doudin K., Patel U., Weston P., Milward M. (2022). Discovery, validation, and diagnostic ability of multiple protein-based biomarkers in saliva and gingival crevicular fluid to distinguish between health and periodontal diseases. J. Clin. Periodontol..

[82] Marmo G.M., Munzone M., Polizzi A., Campagna R., Mascitti M., Isola G. (2025). Genetic and Epigenetic Biomarkers for the Early Oral Cancerization Risk in Periodontitis Patients. Curr. Issues Mol. Biol..

[83] Pratitasari E.V., Nugraha D.A., Rahmat N. (2025). Recent Advances in Digital Saliva Biosensors for Point-of-Care Testing and Periodontitis Monitoring: A Narrative Review. World J. Adv. Res. Rev..

[84] Yaghmoor W.E. (2025). Salivary Biomarkers for Early Detection of Periodontal Diseases: A Systematic Review. J. Contemp. Dent. Pract..

[85] Kikuchi T., Hayashi J., Mitani A. (2022). Next-Generation Examination, Diagnosis, and Personalized Medicine in Periodontal Disease. J. Pers. Med..

